# Tele–Mental Health for Reaching Out to Patients in a Time of Pandemic: Provider Survey and Meta-analysis of Patient Satisfaction

**DOI:** 10.2196/26187

**Published:** 2021-07-29

**Authors:** Raffaele Mazziotti, Grazia Rutigliano

**Affiliations:** 1 Institute of Neuroscience National Research Council (CNR) Pisa Italy; 2 Department of Neuroscience, Psychology, Drug Research and Child Health (NEUROFARBA) University of Florence Florence Italy; 3 Department of Pathology University of Pisa Pisa Italy

**Keywords:** telepsychiatry, telepsychology, e-mental health, document clustering, survey, COVID-19, access to care, patient satisfaction, mental health, tele–mental health, review, telemedicine, satisfaction, access

## Abstract

**Background:**

The COVID-19 pandemic threatened to impact mental health by disrupting access to care due to physical distance measures and the unexpected pressure on public health services. Tele–mental health was rapidly implemented to deliver health care services.

**Objective:**

The aims of this study were (1) to present state-of-the-art tele–mental health research, (2) to survey mental health providers about care delivery during the pandemic, and (3) to assess patient satisfaction with tele–mental health.

**Methods:**

Document clustering was applied to map research topics within tele–mental health research. A survey was circulated among mental health providers. Patient satisfaction was investigated through a meta-analysis of studies that compared satisfaction scores between tele–mental health and face-to-face interventions for mental health disorders, retrieved from Web of Knowledge and Scopus. Hedges g was used as the effect size measure, and effect sizes were pooled using a random-effect model. Sources of heterogeneity and bias were examined.

**Results:**

Evidence on tele–mental health has been accumulating since 2000, especially regarding service implementation, depressive or anxiety disorders, posttraumatic stress disorder, and special populations. Research was concentrated in a few countries. The survey (n=174 respondents from Italy, n=120 international) confirmed that, after the onset of COVID-19 outbreak, there was a massive shift from face-to-face to tele–mental health delivery of care. However, respondents held skeptical views about tele–mental health and did not feel sufficiently trained and satisfied. Meta-analysis of 29 studies (n=2143) showed that patients would be equally satisfied with tele–mental health as they are with face-to-face interventions (Hedges *g*=−0.001, 95% CI −0.116 to 0.114, *P*=.98, *Q*=43.83, *I*^2^=36%, *P*=.03) if technology-related issues were minimized.

**Conclusions:**

Mental health services equipped with tele–mental health will be better able to cope with public health crises. Both providers and patients need to be actively engaged in digitization, to reshape their reciprocal trust around technological innovations.

## Introduction

In March 2020, a COVID-19 outbreak spread throughout the globe reaching the size of a pandemic. Most governments responded with physical distancing measures. In this science fiction–like context, mental health is expected to pay a heavy toll [1]. Paradoxically, in a time of increased mental health vulnerability, access to care had to be restricted by pausing nonurgent outpatient services, closing day centers, and reducing home visits [2-6]. There was one recourse to address reduced access to care—tele–mental health was used to reach out and support patients [7-9]. In most health care organizations, personnel had limited previous experience, and there were little or no regulations in place [10].

**Figure 1:**
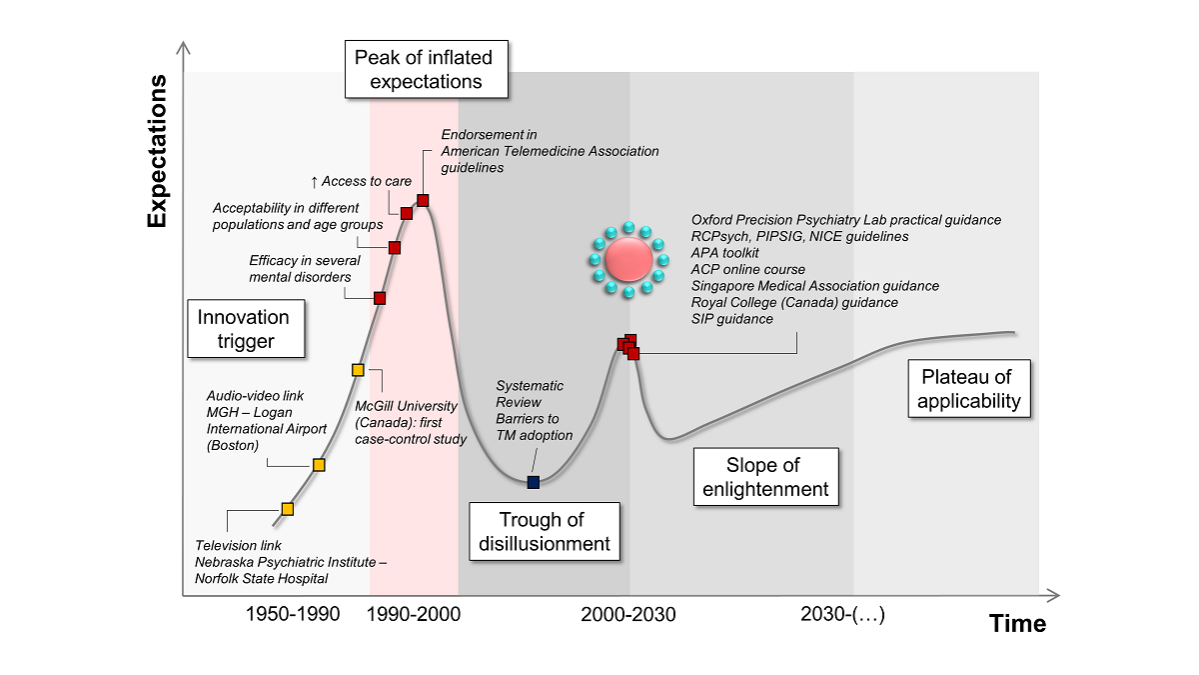
The double-peak effect Gartner Hype Cycle describes the course of tele–mental health, integrating the COVID-19 pandemic as a special or unusual circumstance.

The term *tele–mental health* refers to the remote delivery of mental health care using telecommunications, such as telephone, email, interactive video, digital imaging, and health care monitoring devices [10-12]. The evolution of tele–mental health can be modeled with the double-peak effect Gartner Hype Cycle [13], which describes the course of new technological discoveries integrating special or unusual circumstances (ie, the COVID-19 pandemic) (Figure 1). The Gartner Hype Cycle has served as a useful descriptive model in other medical fields, such as the ultra-high risk for psychosis paradigm [14,15]. According to the Gartner Hype Cycle, new technologies trigger inflated expectations in the short term, and in the long term, expectations are largely underestimated. For tele–mental health, the innovation trigger (stage 1) was the set-up, in 1959, of the first television links between the Nebraska Psychiatric Institute and the Norfolk State Hospital for providing therapy, consultation-liaison psychiatry, and medical student training. Over subsequent years, tele–mental health became increasingly common (stage 2, inflated expectations), expanding in scope to several diagnostic and therapeutic applications, and geographically, from the United States to other countries, in particular, to Australia and Canada. Much enthusiasm developed around tele–mental health’s ability to reach remote rural areas, which suffer from systemic mental health care shortages. By the 2000s, evidence on the use of tele–mental health had accumulated, demonstrating its (1) validity and efficacy in several mental disorders, (2) applicability to different patient populations (eg, war veterans, comorbid medical conditions) and age groups, (3) versatility (diverse cultures), and (4) ability to increase access to care [16]. Despite encouraging evidence and endorsement in clinical guidelines, the adoption of tele–mental health has been slow and scattered, owing to several barriers from clinicians’ perspectives, such as concerns regarding ability to establish a good doctor–patient rapport, confidentiality and data protection, safety, technology-related factors, and financial and legal aspects (stage 3, trough of disillusionment) [17]. The COVID-19 crisis has boosted the attention paid to tele–mental health. In an incredibly short time, a broad array of educational resources, toolkits, and guidelines have been made available. Mental health professionals from around the globe have joined forces and shared their experiences in an effort to provide the best care to patients during this terrible time. The digitization of the field of medicine has become a matter of public interest (stage 4, slope of enlightenment). We will find out, in the years to come, whether this unexpected massive public effort will crystallize into mental health service organization and resource allocation (stage 5, plateau of applicability).

In this study, we provide an analysis of state-of-the-art scientific publications on tele–mental health by applying document clustering to map prominent research topics in the field. We surveyed mental health professionals about their experiences of care delivery during the pandemic, especially regarding their use of and attitudes toward tele–mental health. Patients’ perspectives on tele–mental health were assessed through a systematic review and meta-analysis of satisfaction with tele–mental health compared to face-to-face interventions.

## Methods

### Analysis of Scientific Publications

#### Data Collection

The publication search was performed using Scopus advanced search [18], with the following search formula:

TITLE-ABS-KEY ( telepsychiatry ) OR TITLE-ABS-KEY ( telepsychology ) OR TITLE-ABS-KEY ( telepsychotherapy ) AND ( LIMIT-TO ( PUBSTAGE , “final” ) ) AND ( LIMIT-TO ( DOCTYPE , “ar” ) ) AND ( LIMIT-TO ( SRCTYPE , “j” ) ) AND ( LIMIT-TO ( LANGUAGE , “English” ) )”.

We restricted the search to articles describing original research performed in the field of tele–mental health, while excluding review papers. Results (653 articles on June 4, 2020) were exported in .csv format, with as much information as possible.

#### Data Analysis

After excluding 212 articles for having no relation with tele–mental health (manual filtration) or no abstract, the remaining corpus of 441 articles was imported to Python (version 3; Pandas package, version 1.2.3). Abstracts and titles were concatenated and tokenized (NLTK package, version 3.5). After part-of-speech tagging (filtering only nouns, adjectives and verbs) and lemmatization, common stop words were removed, and stemming was performed. We calculated bigrams (gensim, version 3.8.1) and subsequently removed an array of stop words with broad meanings, such as “paper,” “method,” “analyze,” and other terms that appear in almost every paper. Each tokenized abstract was transformed into a numerical multidimensional representation (TfidfVectorizer, version 0.22.1), which transforms the tokens into an array of term frequency–inverse document frequency values. The similarity between documents was computed with cosine distance between term frequency–inverse document frequency vectors and visualized with *t*-distributed stochastic neighbor embedding, which was used to perform hierarchical density-based spatial clustering of applications with noise [19]. The code is freely available [20].

### Provider Survey

We developed an Italian-language web-based survey targeting mental health providers to map (an English-language version was circulated in an international network of mental health providers.): (1) COVID-19–related disruptions in care provision; (2) tele–mental health use during and prior to the COVID-19 pandemic; (3) tele–mental health intention-to-use; and (4) attitude toward tele–mental health. Sociodemographic (age and gender), employment role, setting, and geographic area information was collected. The survey, designed to be completed within 10 to 15 minutes, consisted of 6 sections, with 21 multiple- or forced-choice questions and 15 Likert-scale questions. The survey was shared through email invitations and social media. The survey remained open for 20 days (from May 30, 2020 to June 20, 2020). All respondents provided informed consent.

### Meta-analysis of Patients’ Satisfaction With Tele–Mental Health Interventions

#### Search Strategy and Selection Criteria

A systematic review and meta-analysis were conducted based on the Population, Intervention, Comparisons, Outcomes and Study Design (PICOS [21]) strategy. We used a 2-step search strategy. First, we searched the Web of Knowledge (Thomson Reuters) and Scopus databases, using the following terms:

(telepsychiatry OR telepsychiatric OR telepsychology OR teletherapy OR tele–mental health OR e-mental) AND (satisfaction).

The search was extended until June 10, 2020. Second, we implemented an electronic manual search of the reference lists of the retrieved articles. Duplicate references were manually removed. Articles were screened by title and abstract, and the full-texts of remaining articles were further inspected for eligibility against a priori defined inclusion and exclusion criteria.

We included original articles written in English that included patients with a diagnosis of any mental disorders and whose study design included both tele–mental health and face-to-face groups that reported satisfaction scores for both groups. Articles were excluded if they only reported data on service acceptability, credibility, and working alliance; failed to report enough data for meta-analysis (authors were contacted to obtain missing data); or presented data from overlapping data sets (in which case, we selected the largest one).

Literature search, study selection, and data extraction were performed by both authors independently. Disagreement was resolved by discussion. The study followed PRISMA (Preferred Reporting Items for Systematic Reviews and Meta-Analyses [21]) guidelines (Table S1 in Multimedia Appendix 1). The protocol was registered in PROSPERO (CRD42020192299).

#### Data Extraction

We extracted author, publication year, setting (country, underserved area), mental disorder diagnosis, population type, study design, intervention type, intervention duration, intervention modality, satisfaction scale, number of participants in the tele–mental health and face-to-face group, age, and gender. As a measure of satisfaction, we extracted mean satisfaction score, standard deviation, or standard error of the mean, *t* test statistic, or *P* value of the *t* test, if the normality assumption was met in the original paper (Methods S1 in Multimedia Appendix 1).

#### Data Analysis

The meta-analysis was performed using R (version 4.0.0, The R Project; meta [22], metaphor [23], dmetar [24] packages). We calculated Hedges *g* and relative standard error. Since high heterogeneity was expected, we pooled effect sizes using a random-effect model [25]. We assessed between-study heterogeneity using the *Q* statistic and quantified total variability using the *I*^2^ index [26]. To assess the robustness of results, we performed influence analyses with graphical display of heterogeneity plots [27], by sequentially fitting our meta-analysis model to all 2*^k^*^−1^ possible combinations of the studies. We applied 3 clustering algorithms—*k*-means, density-based spatial clustering of applications with noise, and the Gaussian mixture model—to detect studies with heavy influences on the overall effect size estimate. Sensitivity analyses were conducted by removing these heavy-influence studies and re-running the meta-analysis (Methods S2 in Multimedia Appendix 1). We performed subgroup analyses with mixed-effect models to determine the influence of predefined categorical moderators: mental disorder diagnosis, population type, underserved area, study design, intervention type, and satisfaction scale. Meta-regression models were fit to investigate the influence of predefined continuous predictors: publication year, mean age, proportion of females, intervention duration, and sample size. We assessed publication bias with the Egger test [28], and risk of bias was examined with the revised Cochrane tool for randomized trials [29].

## Results

### Analysis of State-of-the-Art Scientific Publications

**Figure 2:**
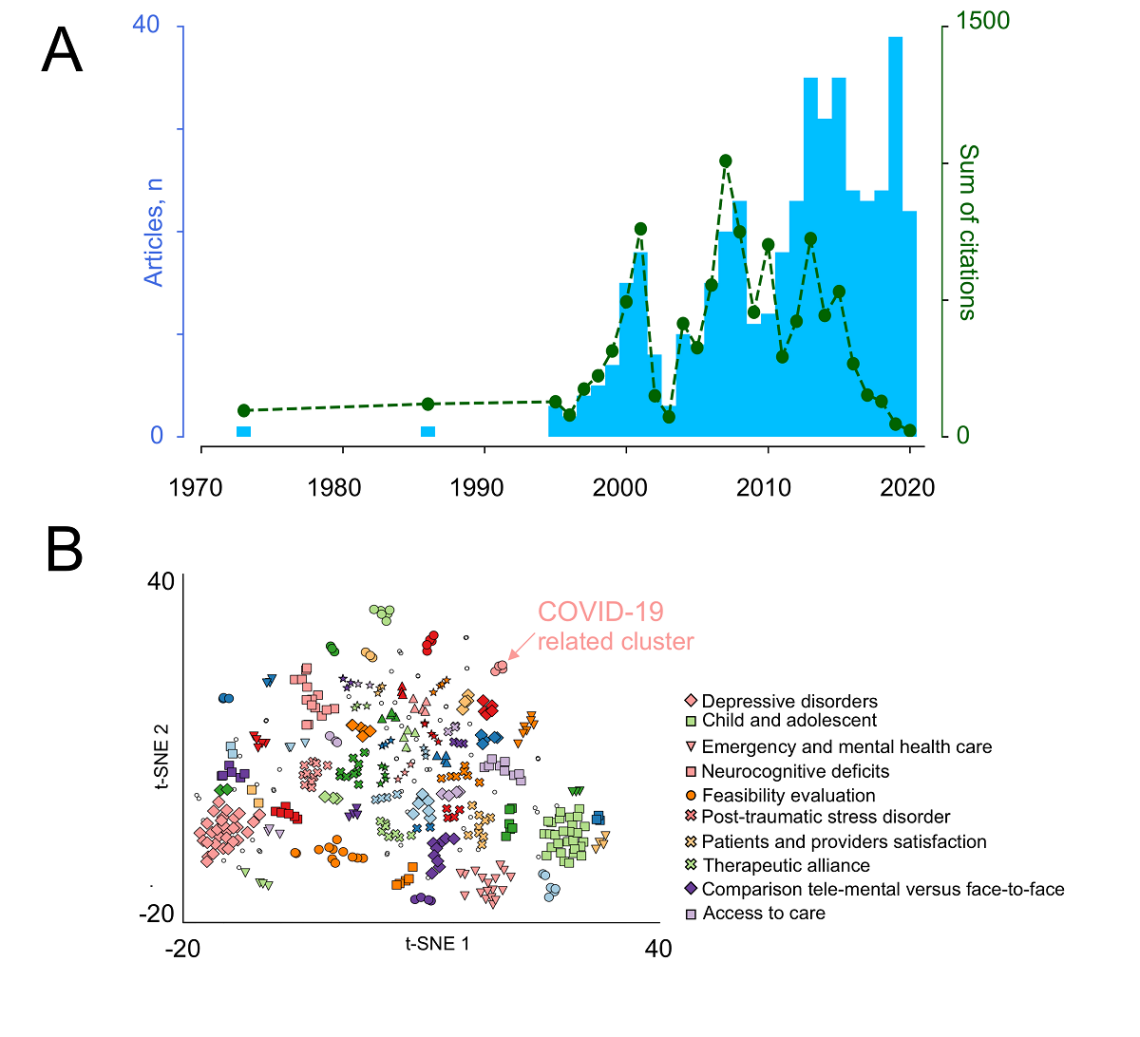
State-of-the-art of tele–mental health scientific publications: (A) Number of articles by year (blue bars) and the sum of citations for annual articles (green dotted line) and (B) Document clustering (total identified topics: 36) showing emerging COVID-19–related topics (pink dots, arrow) in relation to the top 10 topics (legend). PTSD: posttraumatic stress disorder; t-SNE: t-distributed stochastic neighbor embedding.

The field was pioneered in 1973, by a paper published in the *American Journal of Psychiatry*, which described an interactive television system that connected Massachusetts General Hospital and a medical station in Boston (United States) [30]. In 1986, a group from McGill University (Canada) [31] published the first case-control study, which found no substantial difference between tele–mental health and face-to-face in terms of satisfaction among patients and providers. It was only in 1995 that a systematic interest developed, and the annual number of articles began to grow steadily, to reach 39 records in 2019. We expect a further surge in 2020, from a renewed interest for tele–mental health caused by the COVID-19 pandemic. Annual number of articles and sum of citations followed the same pattern until 2010. Then, the sum of citation declined, because more time is required for newly published articles to accumulate citations (Figure 2A).

Only 10 articles had more than 100 citations each (range 120-244; Table S2 in Multimedia Appendix 1). Of these, 1 was the above-mentioned study published in 1986 [31], 8 were published between 2000 and 2010 [32-39], and 1 was published in 2013 [40]. All top-cited articles, except one [37], were controlled trials. The 10 top articles came from only 4 countries—United States, Canada, Australia, and United Kingdom—which are the countries contributing the most to the whole article data set (N=363 articles, 82%; Figure S1A in Multimedia Appendix 1). In the remaining countries, including Italy, that delivered 5 articles or less, tele–mental health research might be at an early stage, corresponding to scarce, if not absent, applications. In terms of international cooperation, the main hub countries are United States, United Kingdom, Australia, and Canada. Other cooperation patterns are more scattered, possibly being more occasional (Figure S1B in Multimedia Appendix 1).

Document clustering identified 36 topics (Figure 2B). The top 10 topics (Table S3 in Multimedia Appendix 1) encompass 34% proportion of the data set. Of these, 6 are specific subjects concerning issues related to the implementation of tele–mental health services. Two topics regard tele–mental health interventions for depressive or anxiety disorders and posttraumatic stress disorder. Finally, 2 topics are focused on the use of tele–mental health in peculiar populations, such as children and adolescents, and patients with neurocognitive deficits (Figure 2B, Figure S1C, and Table S3 in Multimedia Appendix 1).

### Providers’ Responses to the Survey

**Figure 3:**
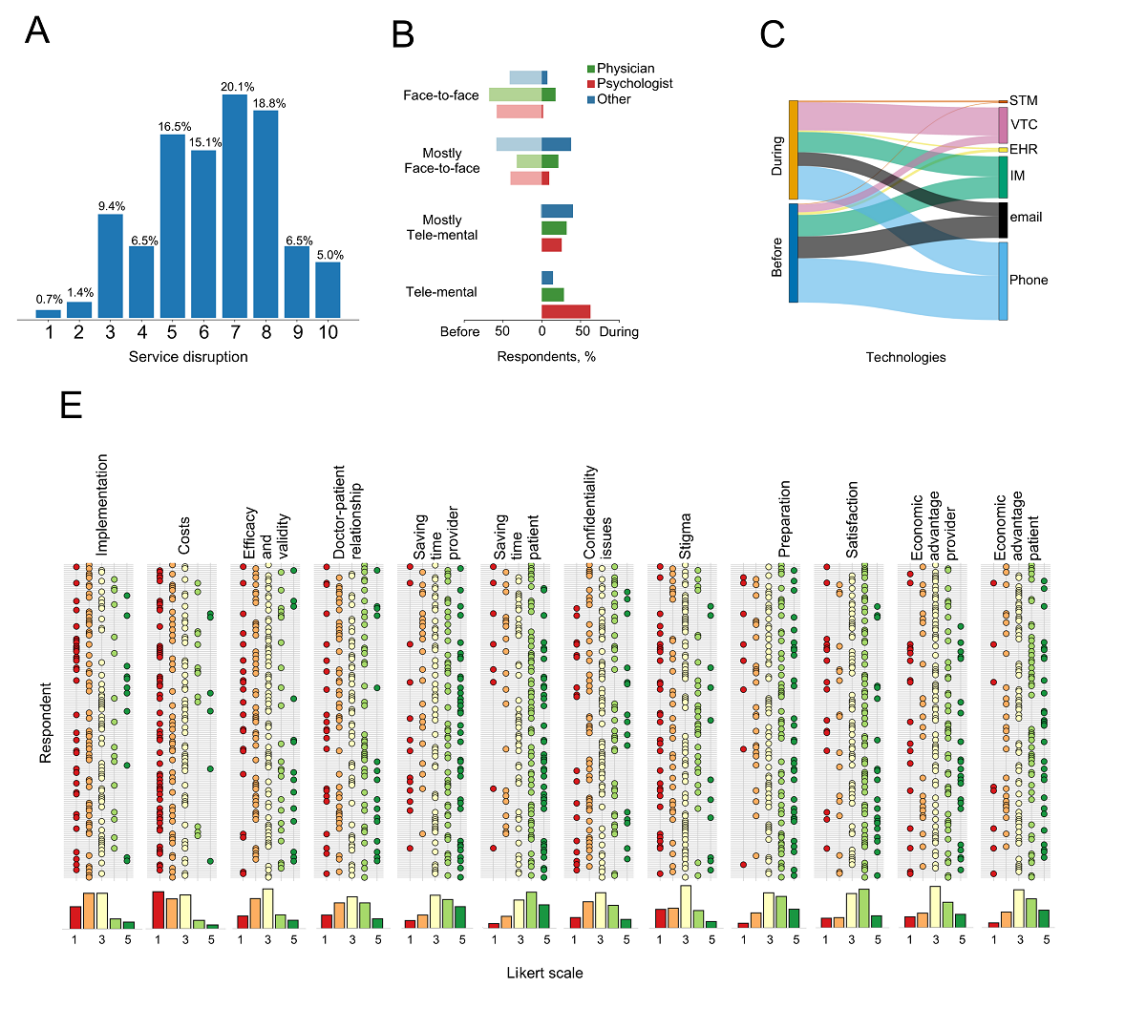
Italian providers’ responses to the survey on the use of tele–mental health during the COVID-19 pandemic: (A) COVID-19–related disruption in mental health service provision; (B) number of physicians, psychologists, and other mental health professionals offering services exclusively face-to-face, mostly face-to-face, mostly by tele–mental health, or exclusively by tele–mental health during and prior to the pandemic; (C) tele–mental health tools used during and prior to the pandemic; and (D) providers’ attitude toward tele–mental health. EHR: electronic health record; IM: instant messaging; STM: supported telemedicine systems; VTC: video-based teleconferencing.

The survey was completed by 174 Italian mental health care providers, 112 (64.4%) of whom were female. Most respondents (75/174, 43.1%) were between 30 and 40 years old. The most represented region was Tuscany (n=42), followed by Lombardy (n=37) and Apulia (n=28) (Figure S2A in Multimedia Appendix 1), and 67.8% of respondents (n=118) were employed in the public sector, including inpatient or outpatient clinics, hospitals, psychiatric residential facilities, residences for the implementation of safety measures, and addiction treatment services, while 56 (32%) worked in the private sector, either in solo or group (n=4) settings. Our sample consisted of 83 (47.7%) physicians, 63 psychologists (36.2%), and 28 (16.1%) other mental health workers, that is, specialized nurses and professionals providing rehabilitative and educational interventions. The majority of the sample (134/174, 77.0%) reported that COVID-19 disrupted their normal service provision. The main reason (55%) was a reduction (or block) in nonurgent services, sometimes accompanied by conversion of structures to COVID clinics. Lockdown was the culprit in 44% of cases, while disruption was directly caused by the virus, that is, being infected or quarantined following contact with someone infected, in 2 cases. The median of COVID-19-related disruption was 7/10 (IQR 5-8) (Figure 3A).

During the pandemic, the rate of respondents providing any services through tele–mental health doubled, passing from 47.7% (83/174) to 92.5% (161/174); 68% respondents reported using mostly or exclusively tele–mental health (vs 1/174, or 0.6%, prior to the pandemic) (Figure 3B). Psychologists reported the highest rate of tele–mental health almost exclusive use (56/63, 89%), compared with psychiatrists (45/83, 54%) and other mental health workers (17/28, 61%) (χ_6_^2^=45.97, *P*<.001) (Figure 3B). Respondents provided a variable amount of care provisions through tele–mental health, in contrast to their previous practice, where tele–mental health was used for less than 25% of care provisions in 82% of cases (Figure S2B in Multimedia Appendix 1). The use of telephone, instant messaging tools, emails remained stable, while we observed a 3.7-fold increase in the use of video-based teleconferencing (Figure 3C). Even if most respondents (132/174, 75.8%) found tele–mental health much or very much useful during the COVID-19 crisis (Figure S2C in Multimedia Appendix 1), 82% (143/174) envisaged to reduce tele–mental health use after the pandemic was over. Half of respondents had to resort to personal telecommunications at their own initiative, as only a small fraction of work settings were adequately equipped (31/118, 26.2% and 19/56, 33.9% in the public and private sector, respectively); however, an effort was made by the Italian National Health System to strengthen tele–mental health during the crisis, as reported by 28% of those in public work settings.

Depression and anxiety disorders were deemed amenable to tele–mental health interventions by a large portion (70/174, 40.2%) of our sample. On the contrary, schizophrenia and other psychotic disorders, substance use disorders and major neurocognitive disorders ranked low (Figure S2D in Multimedia Appendix 1). One-third of respondents (50/174, 28.7%) considered tele–mental health particularly useful for underserved populations, but 25% (44/174) would offer tele–mental health to any population group (Figure S2E in Multimedia Appendix 1).

Providers’ global attitude was skeptical. Only 21.3% of respondents (37/174) thought that tele–mental health was as valid, accurate and effective as face-to-face; 66.1% (115/174) were not positive about the ability to establish a good doctor–patient relationship. Most (132/174, 75.9%) did not believe that tele–mental health could reduce the barrier of stigma. Less than half of respondents felt somewhat or very much trained and satisfied with tele–mental health (Figure 3D).

We received 120 responses from mental health providers from the 5 continents, which replicated Italian data, except for a more positive attitude toward tele–mental health interventions, in terms of: ability to reduce stigma (70/120, 58.3%); feeling prepared and satisfied with tele–mental health care (91/120, 75.8% and 80/120, 66.7%, respectively) (Results S1, Figures S3 and S4 in Multimedia Appendix 1).

### Meta-analysis of Patient Satisfaction With Tele–Mental Health Interventions

Eligibility screening of 247 articles yielded 41 articles (Figure S5 in Multimedia Appendix 1). Of these, 12 could not be included in the meta-analysis because they did not report enough data for computation (Table S4 in Multimedia Appendix 1). The final sample included 29 studies (Table 1), contributing data from 2143 patients (tele–mental health: n=1039; face-to-face: n=1104; 34% female), with mean age of 39.4 years (SD 14.3). The average sample size was 74 (range 12 to 254). The majority (n=19) of studies were conducted in the United States. Approximately half (n=13) of studies reported on tele–mental health in remote geographic areas, such as Thunder Bay in Canada [33,41], the Hawaiian Islands [42-44] and Pacific northwest of the United States [45], rural Australia [46], or targeted underserved communities, such as Native American communities [47], Hispanic communities [48], low-income patients with HIV [49], and inmates of correctional institutions [50-52]. The most represented diagnosis was depression or anxiety disorders (n=11), followed by posttraumatic stress disorder (n=6), alcohol or substance use disorders (n=3), and attention deficit hyperactivity disorder or disruptive disorders (n=2); 7 studies included individuals with any mental disorder. Thirteen studies recruited only adult individuals, while 3 recruited children or adolescents and their caregivers. Most studies offered services to special populations, such as military personnel or veterans (n=10) and individuals in correctional settings (n=3). Eleven studies used telepsychiatry (8 providing consultations and 3 assessment), 17 studies used telepsychology or counseling, and 1 study used both; most studies (n=24) were randomized controlled trials. Mean follow-up was 227 days (range 90 to 540 days) for telepsychiatry and 70 days for telepsychology. The preferred modality was video-based teleconferencing (n=24), the rest were telephone- or web-based interventions. Patient satisfaction was assessed with standardized validated scales in 21 studies; 8 studies used custom tools.

**Table 1:** Studies included in the meta-analysis.

Diagnosis, study type		Population	Age^a^	F (%)^b^	Intervention^c^	Measure	Tele–mental health, n/N (%)	Face-to-face, n/N (%)	Hedges *g* (95% CI)
**Attention-deficit hyperactivity disorder**									
	RCT^d^ [45]	Children and caregivers	9.3	30	Caregiver behavior training; 25 weeks	CSQ^e^-ADHD	—^f^ /12 (—)	—/25 (—)	−0.817 (−1.531 to −0.102)
	RCT [53]	Children and caregivers	10.4	32	Group parent training; 10 weeks	Custom	8/9 (88.9)	11/13 (84.6)	−0.005 (−0.916 to 0.906)
**Alcohol use disorder**									
	Pilot [54]	Volunteers	36.6	43	2 motivational interviews (phone also)	Custom	20	10	0.005 (−0.754 to 0.764)
**Any mental disorder**									
	RCT [55]	Patients (severe)	46.3	48	Telepsychiatry; 18-month follow-up	GGZ^g^	33/47 (70.2)	38/46 (82.6)	0.495 (0.022 to 0.969)
	RCT, eq [33]	Outpatients	—	63	Telepsychiatry; 4-month follow-up	CSQ-8	125/241 (51.9)	129/254 (50.8)	−0.051 (−0.297 to 0.195)
	Comp^h^, pilot [46]	Children and adolescents	12.3	28	Telepsychiatry	Survey	13	34	0.004 (−0.635 to 0.643)
	Test–retest [47]	American Indian veterans	54	0	Telepsychiatry	Custom	53	53	−0.112 (−0.493 to 0.269)
	Comp [50]	Forensic psychiatric patient inmates	34.2	—	Interview	Survey^i^	23	20	0.000 (−0.599 to 0.599)
	Comp [52]	Correctional institution inmates	31.8	0	Telepsychiatry or telepsychology	CSQ-8	86	100	−0.081 (−0.369 to 0.207)
	RCT [51]	Forensic psychiatric patient inmates^j^	42	43	Competency test^k^	Custom	11	10	−0.173 (−1.031; 0.685)
**Major depressive disorder**									
	RCT [34]	Veterans	49.7	12	Telepsychiatry; 6-month follow-up	Custom	43/59 (72.9)	42/60 (70)	0.280 (−0.148 to 0.707)
	RCT, pilot [41]	Outpatients	—	62	Telepsychiatry; 4-month follow-up	CSQ-8	8/12 (66.7)	9/12 (75)	−1.142 (−2.177 to −0.108)
	RCT [48]	Low-income Hispanic patients	43	88	Telepsychiatry; 6-month follow-up	VSQ^l^-9	69/80 (86.3)	78/87 (89.7)	0.166 (−0.159 to 0.490)
	RCT, pilot [49]	Low-income patients with HIV	45.1	74	CBT^m^ (phone only); 14 weeks	SIMH^n^	14/16 (87.5)	17/18 (94.4)	−0.354 (−1.067 to 0.359)
	RCT, prag [56]	Outpatients	43.1	66	Psychotherapy and web-based; 12 weeks	CSQ-8	37/51 (72.5)	32/47 (68.1)	0.071 (−0.402 to 0.544)
	RCT [57]	General	35.6	71	Telepsychiatry; 12-month follow-up	CSQ-8	45/53 (84.9)	40/54 (74.1)	−0.443 (−0.874 to −0.012)
	RCT, NI [58]	Older adult veterans	63.9	2.5	Behavior activation; 8 weeks	CPOSS-VA^o^	100/120 (83.3)	104/121 (86)	−0.256 (−0.531 to 0.020)
	RCT, NI [59]^p^	Military and veterans	—	18	Behavior activation; 8 weeks	CSQ-8	45/62 (72.6)	42/59 (71.2)	−0.142 (−0.563 to 0.279)
**Mood or anxiety disorders**									
	RCT [60]	Outpatients	30	58	CBT; 12 weeks	CSQ	13/14 (65)	11/12 (91.7)	0.115 (−0.689 to 0.919)
**Obsessive compulsive disorder**									
	RCT, NI [61]	Outpatients	31.9	60	CBT (phone only); 10 weeks	CSQ	34/36 (94.4)	32/36 (88.9)	−0.331 (−0.818 to 0.155)
**Opioid use disorder**									
	RCT, pilot [62]	In treatment (methadone)	40.6	62	Web-based^q^ counseling; 6 weeks	Survey	20/33 (60.6)	17/17 (100)	0.004 (−0.642 to 0.651)
	RCT [63]	In treatment (drug abstinence)	41	56	Web-based^q^ counseling; 12 weeks	CSQ-8	22/50 (44)	35/35 (100)	0.437 (−0.103 to 0.976)
**Posttraumatic stress disorder**									
	RCT, NI [38]	Veterans	55.5	0	Group^r^ CBT; 14 weeks	CPOSS-VA	9/17 (52.9)	12/21 (57.1)	0.247 (−0.621 to 1.115)
	RCT, NI [42]	Veterans	55.1	0	Anger management group; 12 weeks	CPOSS-VA	55/61 (90.2)	57/64 (89.1)	−0.015 (−0.386 to 0.355)
	RCT, pilot [43]	Veterans	—	0	Coping skills group; 8 weeks	Custom	8/9 (88.9)	4/8 (50)	0.405 (−0.808 to 1.618)
	RCT, NI [44]	Veterans	55.3	0	Cognitive processing therapy	CPOSS-VA	46/61 (75.4)	50/64 (78.1)	0.249 (−0.153 to 0.651)
	RCT [64]	Veterans	50.0	0	Telepsychiatry^s^; 3-month follow-up	Custom	30/37 (81.1)	30/34 (88.2)	0.888 (0.357 to 1.419)
	RCT, NI [65]	Veterans	44	6	Prolonged exposure; 12 weeks	CPOSS-VA	27/75 (36)	40/75 (53.3)	−0.056 (−0.544 to 0.432)
**Social phobia**									
	RCT [66]	Volunteers	24.4	—	Web-based^t^; 2 months	Custom	—/30 (—)	—/22 (—)	0.104 (−0.446; 0.655)

^a^Mean, in years.

^b^Percentage of female individuals included in each study.

^c^The intervention used video-based teleconferencing, unless otherwise indicated.

^d^RCT: randomized controlled trial—eq indicates equivalence, NI indicates noninferiority, and prag indicates pragmatic.

^e^CSQ: Client Satisfaction Questionnaire—8, 9, and ADHD indicate the 8-item, 9-item, and attention-deficit hyperactivity disorder versions, respectively.

^f^Data were not provided.

^g^GGZ Thermometer.

^h^Comp: comparative study.

^i^Group Health Association of America Consumer Satisfaction Survey.

^j^Patients with Schizophrenia spectrum disorders and mental retardation.

^k^Georgia Court Competency Test Mississippi State Hospital revision.

^l^VSQ: Visit-specific Satisfaction Questionnaire.

^m^CBT: cognitive behavioral therapy.

^n^SIMH: Satisfaction Index Mental Health.

^o^CPOSS-VA: Charleston psychiatric outpatient satisfaction scale: Veteran Affairs version.

^p^Minor depressive disorder was also included.

^q^Getgoing program.

^r^Social and emotional rehabilitation.

^s^Imo voice calls, text messaging, Telegram, and Skype.

^t^Talk to me, a self-administered program.

Our meta-analysis revealed no difference in patient satisfaction between tele–mental health and face-to-face interventions (Hedges *g*=−0.001, 95% CI −0.116 to 0.114, *P*=.985; Figure S6 in Multimedia Appendix 1). There was moderate between-study heterogeneity (*Q*=43,83, *I*^2^=36%, *P*=.03).

The graphical display of heterogeneity plot formed a symmetric distribution, around *g*=0, slightly deviating toward a pattern with positive effect sizes and moderate heterogeneity (peak around 50%) (Figure S7 in Multimedia Appendix 1). Clustering algorithms detected one study [64] that explained the shift toward higher heterogeneity estimates (Figures S7 and S8 in Multimedia Appendix 1). After removing this study, heterogeneity became nonsignificant (*Q*=32.51, *I*^2^=17%, *P*=.214); however, the overall effect size, though slightly more negative (ie, favoring face-to-face over tele–mental health), was not impacted (Hedges *g*=−0.032, 95% CI −0.132 to 0.068, *P*=.531; Figure S11 in Multimedia Appendix 1). These findings were corroborated by other influence diagnostics (Results S2, Figures S9 and S10 in Multimedia Appendix 1).

**Figure 4:**
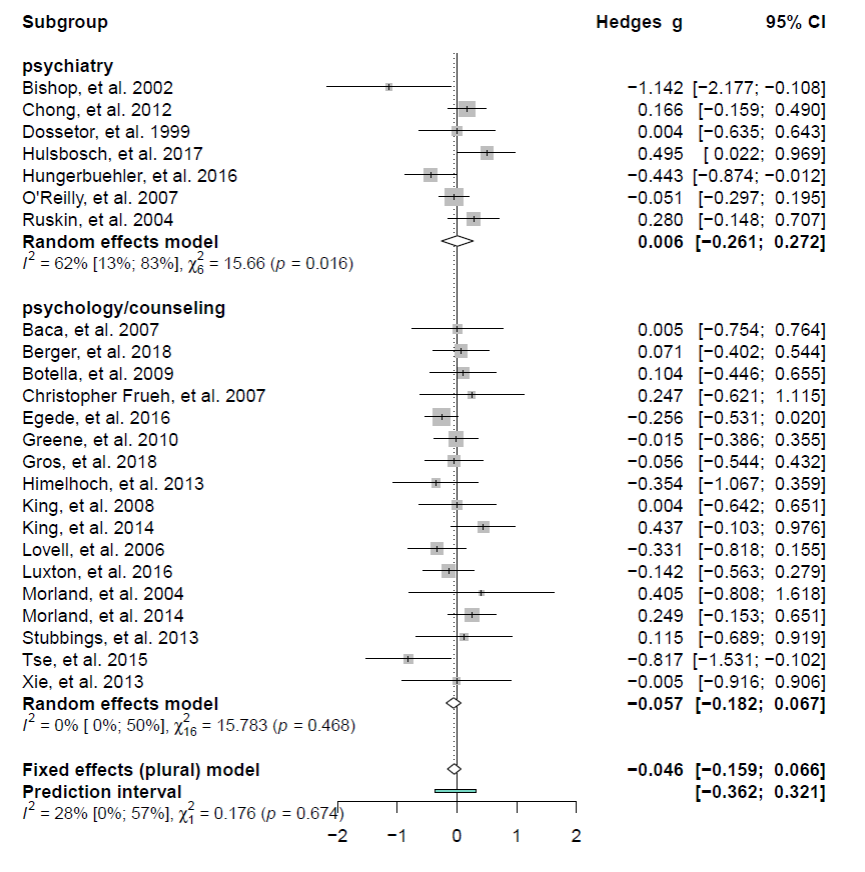
Forest plot of satisfaction scores for tele–mental health vs face-to-face interventions according by intervention type. Positive values favor tele–mental health, while negative values favor face-to-face.

Subgroup analyses, performed after removing [64], did not show any significant differences between mental disorder diagnoses (*P*=.341), population types (*P*=.813), served vs underserved area (*P*=.683), study designs (*P*=.392), and satisfaction scales (*P*=.407) (Figures S12-16 in Multimedia Appendix 1). No significant difference emerged between telepsychiatry vs telepsychology studies, excluding studies providing assessment (total between group heterogeneity *Q*=0.176, *df*=1, *P*=.674). While there was virtually no heterogeneity among telepsychology studies (n=17, *Q*=15,66, *I*^2^=0%, *P*=.468), we found moderate to substantial heterogeneity among telepsychiatry studies (n=7, *Q*=15.78, *I*^2^=62%, *P*<.05) (Figure 4). None of the meta-regression models yielded significant results (publication year: *P*=.417; age: *P*=.207; gender: *P*=.433; intervention duration: *P*=.531; sample size: *P*=.588) (Figures S17-21 in Multimedia Appendix 1).

No publication bias was detected (*t*=0.17, *P*=.867; Figure S22 in Multimedia Appendix 1). There was a high risk of bias for 9 studies, some concerns for 14 studies, and low risk for 6 studies (Figure S24 in Multimedia Appendix 1). The main weakness was due to missing outcome data, as satisfaction scores were generally available for a fraction of randomized participants, which ranged from 36% [65] to 100% [62,63] and varied between intervention arms in the same study (eg, 44% vs 100% in the tele–mental health and face-to-face arms, respectively [63]) (Figures S23 and S24 in Multimedia Appendix 1).

## Discussion

The field of tele–mental health has been continuously evolving since 2000. Such progress was limited to a few countries, namely United States, United Kingdom, Australia and Canada. This might be related to uneven incomes and scientific or technological levels among countries. In addition, tele–mental health may represent a valuable approach to overcome “the tyranny of distance” [46] in countries where substantial portions of the population live in remote rural areas and have unequal access to care. We found that a large amount of tele–mental health literature evaluates (1) service-centered parameters, such as feasibility, acceptability, and sustainability, and (2) care-centered parameters, such as therapeutic alliance, treatment outcome, and patient satisfaction. Depression and posttraumatic stress disorder stand out among the top 10 research topics. Evidence supporting the efficacy of tele–mental health interventions for depression, anxiety, and posttraumatic stress disorders is abundant and robust [67-69]. Another prominent research topic is children and adolescent, a population considered more suited to tele–mental health since they are perceived as digital natives. Two meta-reviews showed that tele–mental health is a valid option for youth with depression and anxiety, while its clinical benefits for autism spectrum disorder, attention deficit hyperactivity disorder, psychosis, and eating disorders remain questionable [70,71]. Tele–mental health has been proven to alleviate pressure on emergency departments (third research topic) [72,73].

Research in tele-mental health did not translate into policy changes and resource allocation. The World Psychiatric Association–Lancet Psychiatry Commission on the Future of Psychiatry has defined 6 core considerations to be met for technological innovations to transform health care: (1) patient and clinician engagement; (2) clinical evidence and standards; (3) clinical systems integration; (4) digital trust, ethics, and transparency; (5) interoperability and scalability; (6) data science and methods [74]. At present, tele–mental health has partially met these targets, due to factors related to both clinicians and patients. Clinicians are often reluctant to adopt tele–mental health because of concerns about the ability to establish a satisfying doctor–patient relationship and lack of knowledge of relevant privacy, transparency, and confidentiality issues [75,76]. A digital divide exists among patients, which excludes a large share of them from tele-mental health interventions [74,77].

When COVID-19 started its inexorable march over the planet, very few countries were sufficiently equipped with tele–mental health technologies, trained clinicians, and guidelines [10,78]. Italy was no exception. Less than one-third of respondents deemed their tele–mental health service to be adequate prior to COVID-19. Care provision was massively disrupted. Qualitative reports have been published, that mostly refer to the situation of the Italian National Health System in Lombardy, which was the most affected Italian region [2-6]. All reports agree that COVID-19 initiated an abrupt transition to tele–mental health delivery of care [2,4-6]. We observed that this change was more evident for psychologists, compared with physicians, probably because physicians carry out part of their clinical activity in in-patient units (COVID-positive patients with serious psychiatric conditions were still admitted to hospital wards) [5] and need to perform physical examinations, while psychotherapy may be more easily conducted remotely. We are aware that the relatively small number of respondents (n=174) hampers generalizability of our observations. Nevertheless, our findings were fully replicated by responses of international mental health professionals (Figure S3 in Multimedia Appendix 1). Also, similar trends have been reported by others in several countries. In China, when the novel human coronavirus (SARS-CoV-2) emerged in December 2019, there was rapid implementation of mental health hotlines and hospital tele–mental health services. In some cases, as the West China Hospital of Sichuan University, tele–mental health services collaborated with courier services to deliver medication to patients’ homes [8]. In most European countries there was a boost in tele–mental health use and value, and regulatory barriers were substantially lifted [79,80]. An analysis of electronic health record data showed a substantial shift from face-to-face to tele–mental health contacts in London, United Kingdom after lockdown measures [81]. These findings are paralleled by those in Australia and Africa [82,83]. In May 2020, the American Psychiatric Association surveyed its members on the matter, and responses from 500 American psychiatrists grossly replicated our findings of a major transition to tele–mental health use—in a couple of months the percentage of respondents seeing more than 75% of their patient caseload via tele–mental health increased from 2.1% to 84.7% [84]. In addition, respondents reported that satisfaction was high or very high among patients first assessed via tele–mental health [84], and Sammons et al [85] reported a similar adaptation to COVID-19 among psychologists in the Trust and National Register (United States).

Half of respondents used personal tools on their own initiative. If, from one side, these spontaneous efforts are to be commended because they allow the system to rapidly adjust to unexpected stressors, then from the other, uncoordinated and uneducated use of such tools might increase the risk of breaches in consent processes, privacy, and data protection or may lack appropriate emergency management plans. We advocate that mental health departments be digitized in order to improve their resilience in face of public health emergencies [2]. Such technological leaps will only be successful if complemented with proper training and supported by policy changes. Conveniently, open-access resources have been flourishing during the last months; we recommend the practical guidance developed by the Oxford Precision Psychiatry Lab [12], and the American Psychiatric Association Toolkit [11]. A better understanding of best practices could modify skeptical views such as those we recorded in our survey. It has been reported that clinicians had a gatekeeper role against tele–mental health, and their concerns were mainly related to the ability to build a meaningful doctor–patient relationship [17]. The integration of tele–mental health in mental health care implies a transition from the current centralized model of care to a more distributed model, in order to re-define the equilibrium between clinicians and patients’ responsibilities. Many issues regarding standard of care, access to data, clinicians’ compensation remain open, thus it is no surprise that clinicians may feel uncomfortable. Research that explicitly addresses these issues will be needed. It will be fundamental to ensure that clinicians’ needs and desires are heard effectively. Formal education about the role of technology in care provision will have to be implemented starting from medical school, without neglecting colleagues who may be less familiar with technology because they have practiced for years before the advent of smartphones and their application in health care. Furthermore, the field will need to meet some key requirements to support the transformation. Borrowing from the recommendations issued by the World Psychiatric Association–Lancet Psychiatry Commission [74] and the American Psychiatric Association [84], we maintain that (1) both psychiatrists and patients should be engaged in all phases of tele–mental health development and implementation and not only as final users; (2) patients’ routine screening should include an assessment of digital access, literacy, and comfort, and specialized education, technical support, and internet (or device) access programs should be offered to improve treatment delivery (especially to vulnerable populations, eg, older people, homeless people, asylum seekers); (3) a tele–mental health ethical code will be needed, to reassure patients about confidentiality and safety issues and help them making informed decisions; (4) tele–mental health sustainability and scalability should be promoted, to avoid care fragmentation and abate costs; (5) current regulations should be reviewed regarding remote drug prescription, use of audio-only communications for patients’ assessment and management, and service frequency in in-patient settings and nursing facilities; (6) careful considerations should be made about compensations and national health insurance programs. These considerations apply, not only to tele–mental health, but also, to digital psychiatry. The last decade has witnessed an expansion in smartphone apps, wearable sensors and other technologies for digital phenotyping of patients suffering from mental disorders, which has been accompanied by the growing use of artificial intelligence in health care. This expansion has mainly been driven by the opportunities offered by technological advancement, which often lack adequate scientific and clinical roots. Research, funded by government programs, will be needed [86]: for instance, digitization is one of the pillars of the €750-billion Next Generation European Union plan (equivalent to approximately US $891 billion), which aims to support recovery from the COVID-19 crisis and also to invest in the future and resilience of our society.

To focus on service users, we investigated how tele–mental health compares to face-to-face interventions in terms of patient satisfaction, because this is a crucial influence on treatment outcome, particularly in mental health [87,88]. We performed a systematic review and meta-analysis, which did not detect a significant difference in satisfaction between tele–mental health and face-to-face (*P*=.985). Because studies were moderately heterogeneous, we applied 2 methods to explore heterogeneity. Both methods showed that one study—Haghnia et al 2019 [64], alone—explained much of the heterogeneity. This study was conducted in Iran, whose conditions might be different from high-income countries. The economic impact, difficulties of travelling, and accommodation requirements associated with face-to-face visits might be more burdensome for people living in the Middle East and justify the higher satisfaction scores found in patients treated by tele–mental health [89]. However, this study had marginal influence on the global effect size. Subgroup analyses showed homogeneity among studies focusing on psychotherapy, as opposed to those focusing on telepsychiatry interventions, which yielded substantially heterogeneous effect sizes. Psychiatric consultations are characterized by high variability, consisting of meetings of variable duration, separated by variable intervals, with variable content based on patients’ incidental needs and medication management. On the contrary, psychotherapies are “healing relationships” [90] developing over a series of evenly distributed contacts of preestablished duration that use evidence-based (often manualized) methods [91]. A previous systematic review [88], which compared tele–mental health to face-to-face–delivered psychotherapeutic interventions, similarly found that patients were equally satisfied with both approaches but highlighted limitations (some of which are also relevant to our study). Most studies included in our review were affected by some risk of bias from high attrition rates, which led to small, underpowered sample sizes. Satisfaction scores were available just for the fraction of patients who remained in treatment. It is plausible that dissatisfaction with treatment was responsible for participants dropping out of the studies and becoming unavailable for satisfaction assessment. However, attrition rates in the 2 treatment arms (tele–mental health vs face-to-face) were similar, most likely causing satisfaction score inflation in both arms with negligible impact on the difference. A selection bias could have been introduced even before randomization, since 6 studies [49,56,57,62,63,87] excluded eligible participants who did not have access to computer and internet connection. In 16 studies [33,34,38,41-48,50-53,60] tele–mental health sessions were held in rooms fully equipped with high-definition video-based teleconferencing units and broadband internet access. In 2 studies, tele–mental health interventions were performed at home, but participants were provided with videophones [58] or computers [55] and a dedicated line. Therefore, in most cases, technology-based factors, which contribute to shape patient satisfaction [88], could have been minimized. This limits the generalizability of their results to ecological contexts: (1) many patients may be marginalized due to lack of access to technology and skills; and (2) problems with video definition, audio lag, or connection could dampen the perceived consultation quality. Another limitation is that we only considered overall patient satisfaction. This is a complex clinical outcome that includes several factors related to patient, disease, provider, therapy, environment, and technology [87]. Rohland et al [92] showed that patients rated tele–mental health higher than face-to-face for convenience, ease, technical skills, attention given, and time spent, while face-to-face was preferable to tele–mental health for self-reporting outcome, helpfulness, and eye contact. It has been suggested that tele–mental health patients develop lower levels of therapeutic alliance, resulting in worse continuity of care [38,93,94], but data are still inconclusive. Whether the relative preference for tele–mental health or face-to-face care has an impact on clinical outcomes in specific domains needs to be determined in future longitudinal studies.

In conclusion, evidence for the use of tele–mental health is robust, but it is concentrated in a few countries. The initial enthusiasm around tele–mental health did not translate to clinical application. During the COVID-19 pandemic, many mental health professionals resorted to tele–mental health, not without some aversion, feeling that “they had no other choice [6].” It is advisable that mental health services should become equipped with tele–mental health to increase the ability to efficiently cope with public health crises. We believe that this does not necessarily contradict the preferences of both clinicians and patients for in-person meaningful therapeutic rapports.

## Appendix

Multimedia Appendix 1 Supplementary information.

## Abbreviations

COVID-19: coronavirus disease 2019
HIV: human immunodeficiency virus
