# Supplementary material for: Tele–Mental Health for Reaching Out to Patients in a Time of Pandemic: Provider Survey and Meta-analysis of Patient Satisfaction
Source: JMIR Ment Health. 2021 Jul 29;8(7):e26187. doi: 10.2196/26187 (PMC8323764; doi:10.2196/26187)
Supplement: Multimedia Appendix 1 [file mental_v8i7e26187_app1.docx]

# Supplementary Information

Mazziotti R., Rutigliano G. **Tele–Mental Health for Reaching Out to Patients in a Time of Pandemic: Systematic Review, Meta-analysis of Patient Satisfaction, and Provider Survey**

[Supplementary Information](#_Toc78130164)

[Table S1. PRISMA guidelines for systematic reviews and meta-analysis](#_Toc78130165)

[Methods S1. Data extraction](#_Toc78130166)

[Methods S2. Supplementary influence diagnostics](#_Toc78130167)

[Table S2. Ten top-cited articles about tele-mental health](#_Toc78130168)

[Table S3. Top 10 topics detected by document clustering in the domain of tele-mental health](#_Toc78130169)

[Figure S1. Analysis of state-of-the-art scientific publications on tele-mental health.](#_Toc78130170)

[Figure S2. Italian providers’ responses to the survey on the use of tele-mental health during the COVID-19 pandemic.](#_Toc78130171)

[Results S1. Data from the International survey on tele-mental health use during the COVID-19 pandemic](#_Toc78130172)

[Figure S3. International providers’ responses to the survey on the use of tele-mental health during the COVID-19 pandemic.](#_Toc78130173)

[Figure S4. International providers’ responses: use of tele-mental health during the COVID-19 pandemic across age groups.](#_Toc78130174)

[Figure S5. PRISMA 2009 Flow Diagram](#_Toc78130175)

[Table S4. Characteristics of the eligible studies not included in meta-analysis](#_Toc78130176)

[Figure S6. Forest plot showing the overall effect size for the comparison of satisfaction scores between tele-mental and face-to-face interventions for mental disorders. Positive values favor tele-mental, while negative values favor face-to-face.](#_Toc78130177)

[Figure S7. Influence analysis with the Graphic Display of Heterogeneity (GOSH) plot](#_Toc78130178)

[Figure S8. Graphic Display of Heterogeneity (GOSH) plot, showing effect size-heterogeneity patterns.](#_Toc78130179)

[Results S2. Supplementary influence diagnostics](#_Toc78130180)

[Figure S9. Influence analysis with the Leave-One-Out method](#_Toc78130181)

[Figure S10. Forest plots of the overall effect sizes recalculated with the Leave-One-Out method, ordered by heterogeneity (A) and effect size (B)](#_Toc78130182)

[Figure S11. Forest plot after removal of the detected outlier (Haghnia, et al. 2019)](#_Toc78130183)

[Figure S12. Subgroup analysis for mental disorder diagnosis](#_Toc78130184)

[Figure S13. Subgroup analysis for population type](#_Toc78130185)

[Figure S14. Subgroup analysis for served (no) vs underserved (yes) area or community](#_Toc78130186)

[Figure S15. Subgroup analysis for non-RCT vs RCT study design](#_Toc78130187)

[Figure S16. Subgroup analysis for custom vs standardized satisfaction scale](#_Toc78130188)

[Figure S17. Meta-regression: Publication year](#_Toc78130189)

[Figure S18. Meta-regression: Age](#_Toc78130190)

[Figure S19. Meta-regression: Gender](#_Toc78130191)

[Figure S20. Meta-regression: Intervention duration](#_Toc78130192)

[Figure S21. Meta-regression: Sample size](#_Toc78130193)

[Figure S22. Assessment of small sample publication bias with the Funnel Plot](#_Toc78130194)

[Figure S23. Risk of bias summary: Authors’ judgements about each risk of bias domain for each included study](#_Toc78130195)

[Figure S24. Risk of bias summary: Authors’ judgements about each risk of bias domain for all included study](#_Toc78130196)

[References](#_Toc78130197)

#### Table S1. PRISMA guidelines for systematic reviews and meta-analysis

| **#** | **Section/topic** | **Checklist item and brief description of how the criteria were handled** | **Section, page** |
| --- | --- | --- | --- |
| **TITLE** | | | |
| 1 | Title | *Identify the report as a systematic review, meta-analysis, or both.*  The study has been identified as a systematic review and meta-analysis of patient satisfaction. | Title |
| **ABSTRACT** | | | |
| 2 | Structured summary | *Provide a structured summary including, as applicable: background; objectives; data sources; study eligibility criteria, participants and interventions; study appraisal and synthesis methods; results; limitations; conclusions and implications of key findings; systematic review registration number.*  All relevant information has been included in the abstract. | Abstract |
| **INTRODUCTION** | | | |
| 3 | Rationale | *Describe the rationale for the review in the context of what is already known.*  Patient satisfaction with the treatment is crucial for successful therapeutic relationship and outcome. Technology-related factors could modify patient satisfaction with treatments offered through tele-mental health modalities. It is presently unclear whether patients are as satisfied with tele-mental interventions as with face-to-face care delivery. | Introduction |
| 4 | Objectives | *Provide an explicit statement of questions being addressed with reference to participants, interventions, comparisons, outcomes, and study design (PICOS).*  We tested if satisfaction with treatment was significantly different between patients receiving tele-mental as compared to face-to-face interventions. We included: patients suffering from any mental disorders; any intervention type (both telepsychiatry and telepsychology/counselling); randomized controlled trials (RCT) and cross-sectional observational studies, also if from pilot datasets. We further investigated source of heterogeneity and moderator factors across studies. | Introduction |
| **METHODS** | | | |
| 5 | Protocol and registration | *Indicate if a review protocol exists, if and where it can be accessed (e.g., Web address), and, if available, provide registration information including registration number.*  The protocol has been submitted for registration on PROSPERO (registration number: CRD42020192299) | Methods, Search strategy and selection criteria |
| 6 | Eligibility criteria | *Specify study characteristics (e.g., PICOS, length of follow-up) and report characteristics (e.g., years considered, language, publication status) used as criteria for eligibility, giving rationale.*  We included: a) original published articles written in English, with no restrictions on publication date b) that included subjects with a diagnosis of any mental disorders, c) whose study design included both tele-mental and face-to-face treatment groups, d) that reported data on measures of patient satisfaction for both groups. Articles were excluded if: a) they were abstracts/reviews/non-original data/case reports or series, b) were written in languages other than English, c) reported only data on measures of service acceptability, credibility, working alliance, d) failed to report enough data for meta-analytical computation (authors were contacted to obtain missing data), e) presented data drawn from overlapping datasets. | Methods, Search strategy and selection criteria |
| 7 | Information sources | *Describe all information sources (e.g., databases with dates of coverage, contact with study authors to identify additional studies) in the search and date last searched.*  Two-step search strategy: 1) Web of Knowledge^SM^ database by Thomson Reuters® (which includes Web of Science Core Collection, BIOSIS Citation Index, KCI ‐ Korean Journal Database, MEDLINE, Russian Science Citation Index, and SciELO Citation Index) and Scopus®. The search was extended until June 10th, 2020; 2) electronic manual search of the reference lists of the retrieved articles. | Methods, Search strategy and selection criteria |
| 8 | Search | *Present full electronic search strategy for at least one database, including any limits used, such that it could be repeated.*  The following terms were used: (*telepsychiatry* OR *telepsychiatric* OR *telepsychology* OR  *teletherapy*  OR *telemental* OR *e-mental*) AND (*satisfaction*). | Methods, Search strategy and selection criteria |
| 9 | Study selection | *State the process for selecting studies (i.e., screening, eligibility, included in systematic review, and, if applicable, included in the meta-analysis).*  The identified articles were screened by title and abstract, and the full text of surviving articles were further inspected for eligibility against *a priori* defined inclusion and exclusion criteria. | Methods, Search strategy and selection criteria, Fig S2 |
| 10 | Data collection process | *Describe method of data extraction from reports (e.g., piloted forms, independently, in duplicate) and any processes for obtaining and confirming data from investigators.*  Data extraction was performed by two independent researchers [GR, RM]. Disagreement was resolved through discussion between the two researchers. Authors were contacted to obtain missing data for meta-analytical computation. | Methods, Data extraction |
| 11 | Data items | *List and define all variables for which data were sought (e.g., PICOS, funding sources) and any assumptions and simplifications made.*  Extracted variables: author, publication year, Country, underserved area/community, mental disorder diagnosis, population type, study design, intervention type, intervention duration, intervention modality, satisfaction scale, number of subjects in the tele-mental group, number of subjects in the face-to-face group, age and gender. | Methods, Data extraction |
| 12 | Risk of bias in individual studies | *Describe methods used for assessing risk of bias of individual studies (including specification of whether this was done at the study or outcome level), and how this information is to be used in any data synthesis.*  Risk of bias was assessed with the Revised Cochrane risk-of-bias tool for randomized trials (RoB 2), and the summary presented in plots and discussed against the main outcome. | Methods, Data analysis |
| 13 | Summary measures | *State the principal summary measures (e.g., risk ratio, difference in means).*  Hedges’ g (standardized mean difference). | Methods, Data analysis |
| 14 | Synthesis of results | *Describe the methods of handling data and combining results of studies, if done, including measures of consistency (e.g., I^2^) for each meta-analysis.*  Effect size pooling using a random-effect model with the DerSimonian-Laird estimator. Q statistics, as measure of heterogeneity. I^2^ index, as measure of the percentage of variation across studies that is due to heterogeneity. | Methods, Data analysis |
| 15 | Risk of bias across studies | *Specify any assessment of risk of bias that may affect the cumulative evidence (e.g., publication bias, selective reporting within studies).*  Publication biases according to the small sample bias method, by using the Egger’s test to quantify funnel plot asymmetry. | Methods, Data analysis |
| 16 | Additional analyses | *Describe methods of additional analyses (e.g., sensitivity or subgroup analyses, metaregression), if done, indicating which were pre-specified.*  Influence analyses with the Graphic Display of Heterogeneity (GOSH) plots. Outlier analysis. Sensitivity analysis with leave-one-out method. Subgroup analyses with mixed-effect model to determine the influence of pre-specified categorical moderators. Meta-regression models to investigate the influence of pre-specified continuous predictors. | Methods, Data analysis, supplementary influence diagnostics |
| **RESULTS** | | | |
| 17 | Study selection | *Give numbers of studies screened, assessed for eligibility, and included in the review, with reasons for exclusions at each stage, ideally with a flow diagram.*  All details are depicted in the PRISMA flow-chart, Fig S2, and described in the main text. | Results; Fig S2 |
| 18 | Study characteristics | *For each study, present characteristics for which data were extracted (e.g., study size, PICOS, follow-up period) and provide the citations.*  For included studies, characteristics and citations are listed in Table 1. The characteristics of eligible studies not included in meta-analysis are listed in Table S4. | Results; Table1; Table S4 |
| 19 | Risk of bias within studies | *Present data on risk of bias of each study and, if available, any outcome level assessment (see item 12).*  Risk of bias and implication for the outcome is reported in the main text, and summarized in Fig 5.c. | Results; Fig 5.c; Fig S18 |
| 20 | Results of individual studies | *For all outcomes considered (benefits or harms), present, for each study: (a) simple summary data for each intervention group (b) effect estimates and confidence intervals, ideally with a forest plot.*  Results of individual studies, in terms of Hedges’g, standard error, 95%CI, and weight, are represented in Fig 4 and described in the Result section. | Results; Fig 4 |
| 21 | Synthesis of results | *Present results of each meta-analysis done, including confidence intervals and measures of consistency.*  Results of the meta-analysis, in terms of overall Hedges’g, 95%CI, prediction interval, and measures of consistency, are represented in Fig 4 and described in the Result section. | Results; Fig 4 |
| 22 | Risk of bias across studies | *Present results of any assessment of risk of bias across studies (see Item 15).*  Results of Egger’s test for publication bias are reported in the Result section. Funnel plot are shown in Fig S17. | Results; Fig S17 |
| 23 | Additional analysis | *Give results of additional analyses, if done (e.g., sensitivity or subgroup analyses, meta-regression [see Item 16]).*  Results of influence analysis using the GOSH plot are presented in the Result section, and graphically represented in Fig S3, Fig 5.a. Results of outlier and sensitivity analysis are presented in Results S2 and Fig S4-S6.Results of subgroup analysis for intervention type are presented in the Result section, with corresponding forest plot in Fig 5.b. Results of other subgroup analyses and meta-regressions are reported in Fig S7-S16. | Results; Fig 5.a; Fig 5.b; Fig S3; Results S2; Fig S4-Fig S16. |
| **DISCUSSION** | | | |
| 24 | Summary of evidence | *Summarize the main findings including the strength of evidence for each main outcome; consider their relevance to key groups (e.g., healthcare providers, users, and policy makers).* | Discussion |
| 25 | Limitations | *Discuss limitations at study and outcome level (e.g., risk of bias), and at review-level (e.g., incomplete retrieval of identified research, reporting bias).* | Discussion |
| 26 | Conclusions | *Provide a general interpretation of the results in the context of other evidence, and implications for future research.* | Discussion |
| **FUNDING** | | | |
| 27 | Funding | *Describe sources of funding for the systematic review and other support (e.g., supply of data); role of funders for the systematic review.*  This work is supported by the University of Pisa, PRA 2020-21 to G.R. | Acknoledgments |

### Methods S1. Data extraction

Our primary outcome measures were mean satisfaction scores for both patients offered tele-mental interventions and those offered face-to-face interventions. Sample size and standard deviation (SD) or standard error of the mean (SEM) were also required. If the normality assumption allowed parametric statistics in the original paper, T test or post-hoc analysis significant level (p-value) were extracted alongside with direction of the effect and sample size.

In this case, for the data extraction we adopted the following a priori rules:

- Statistically significant differences in p-value, were implied as 0.05 (where not directly specified)
- Not statistically significant differences in p-value, were implied as 0.99 (where not directly specified)
- Null hypothesis significance tests were considered two tailed if not otherwise specified.

If needed, continuous variables were merged according to the following formulae:

- Merged sample size = $N1 + N2$
- Merged mean = $\frac{N1M1 +N2M2}{N1 + N2}$
- Merged SD = $\sqrt[2]{\frac{(N1-1){SD1}^{2} + (N2-1){SD}^{2} + \frac{N!N2}{N1 + N2} ({M1}^{2} + {M2}^{2} - 2M1M2)}{N1 + N2 -1}}$

where *N* is the sample size, *M* is the mean and *SD* is the standard deviation.

### Methods S2. Supplementary influence diagnostics

To assess the robustness of results, we searched studies with extreme effect sizes, i.e., outliers. Studies were defined outliers if their 95% confidence interval (CI) did not overlap with the pooled effect’s CI (1).

We conducted sensitivity analyses using the Leave-One-Out-method, by sequentially re-running our meta-analysis k - 1 times, each time removing one study.

We used the function *InfluenceAnalysis* in the *dmetar* R package, to identify studies that influenced and potentially distorted the pooled effect size.

The following parameters of the influence analyses were plotted:

- Standardized residuals: a measure of how much the predicted pooled effect changes after excluding that study;
- dffits: a value that indicates in SD how much the predicted pooled effect changes after excluding that study;
- Cook’s distance: the distance between the value when that study is included compared to when it is excluded;
- Covariance ratio: the ratio between the determinant of the variance-covariance matrix of the parameter estimates when that study is excluded, and the determinant if the variance-covariance matrix of the parameter estimates when all studies are included;
- tau^2^;
- Q;
- hat matrix and leverages: used to identify the pooled effect after study removal that have outlying values for the predicted pooled effect;
- weight.

The *InfluenceAnalysis* function is implemented with the cut-offs proposed by Viechtbauer and Cheung to determine influential studies (1).

We also plotted each study influence on the pooled effect size (y-axis) against its contribution to the overall heterogeneity (x-axis) in the so-called Baujat Plot (2). The Baujat plot allows to identify the studies explaining the heterogeneity in our estimates (falling in the right side of the plot), at the same time showing their impact on the overall pooled effect.

#### Table S2. Ten top-cited articles about tele-mental health

| **Title** | **1^st^ Author** | **Year** | **Country** | **Source title (CiteScore 2019)*** | **Total citations** | **References** |
| --- | --- | --- | --- | --- | --- | --- |
| Internet treatment for depression: A randomized controlled trial comparing clinician vs. technician assistance | Titov, N. | 2010 | Australia | PLoS ONE (5,2) | 244 | (3) |
| Is telepsychiatry equivalent to face-to-face psychiatry? Results from a randomized controlled equivalence trial | O’Reilly, R. | 2007 | Canada | Psychiatric Services (4,2) | 203 | (4) |
| Treatment outcomes in depression: Comparison of remote treatment through telepsychiatry to in-person treatment | Ruskin, P.E. | 2004 | USA | American Journal of Psychiatry (21,9) | 197 | (5) |
| A randomized, controlled trial of child psychiatric assessments conducted using videoconferencing | Elford, R. | 2000 | Canada | Journal of Telemedicine and Telecare (4,9) | 161 | (6) |
| A randomized trial of telemedicine-based collaborative care for depression | Fortney, J.C. | 2007 | USA | Journal of General Internal Medicine (4,2) | 159 | (7) |
| Resisting and promoting new technologies in clinical practice: The case of telepsychiatry | May, C. | 2001 | UK | Social Science and Medicine (5,7) | 150 | (8) |
| A randomized trial of telepsychiatry for post-traumatic stress disorder | Frueh, B.C. | 2007 | USA | Journal of Telemedicine and Telecare (4,9) | 139 | (9) |
| Patients' depression treatment preferences and initiation, adherence, and outcome: A randomized primary care study | Raue, P.J. | 2009 | USA | Psychiatric Services (4,2) | 136 | (10) |
| Improving Adherence and Clinical Outcomes in Self-Guided Internet Treatment for Anxiety and Depression: Randomised Controlled Trial | Titov, N. | 2013 | Australia | PLoS ONE (5,2) | 121 | (11) |
| Telepsychiatry: Psychiatric consultation through two-way television. A controlled study | Dongier, M. | 1986 | Canada | Canadian Journal of Psychiatry (6,8) | 120 | (12) |

* CiteScore is a metric extracted from Scopus

#### Table S3. Top 10 topics detected by document clustering in the domain of tele-mental health

| **Suggested topic** | **Discriminating stemmed terms** | **%** |
| --- | --- | --- |
| Depressive disorders | depress; controlled studi; outcom; anxieti; follow up; treatment outcom; major depress; adher; patient satisfact; patient compli | 6,8 |
| Child and adolescent | child; adolesc; mental health servic; child psychiatri; patient satisfact; teleconsult; remote consult; mental diseas; satisfact; evalu | 6,3 |
| Emergency mental health care | emerg; depart;adolesc; organization and manag; remot; young adult; rural popul; first;mental health servic; health services access | 4,0 |
| Neurocognitive deficits | cognit; neuropsycholog; test; popul; evalu; face to fac; examin; function; analysi; dementia | 3,4 |
| Feasibility evaluation | dementia; controlled studi; follow up; nurs; experi; depress; home; geriatr; satisfact; teleconsult | 2,7 |
| PTSD | ptsd; stress; posttraumat; posttraumatic stress disord; cognitive therapi; treatment outcom; stress disorders, post traumat;controlled studi; cognit; follow up | 2,4 |
| Patients and providers satisfaction | satisfact; patient satisfact; mental health servic; health services access; rural health car; teleconsult; total; telepsychiatr; evalu; face to fac | 2,0 |
| Therapeutic alliance | therapeut; client; allianc; rapport; therapist; session; in person; satisfact; psychologist; condit | 2,0 |
| Comparison tele-mental vs face-to-face | face to fac; satisfact; follow up; evalu; outcom; mental health servic; remote consult; controlled studi; agreement; face face | 2,0 |
| Access to care | mental health servic; urban; rural popul; rural health car; rural health; adolesc; organization and manag; young; underserv; develop | 2,0 |

#### Figure S1. Analysis of state-of-the-art scientific publications on tele-mental health.


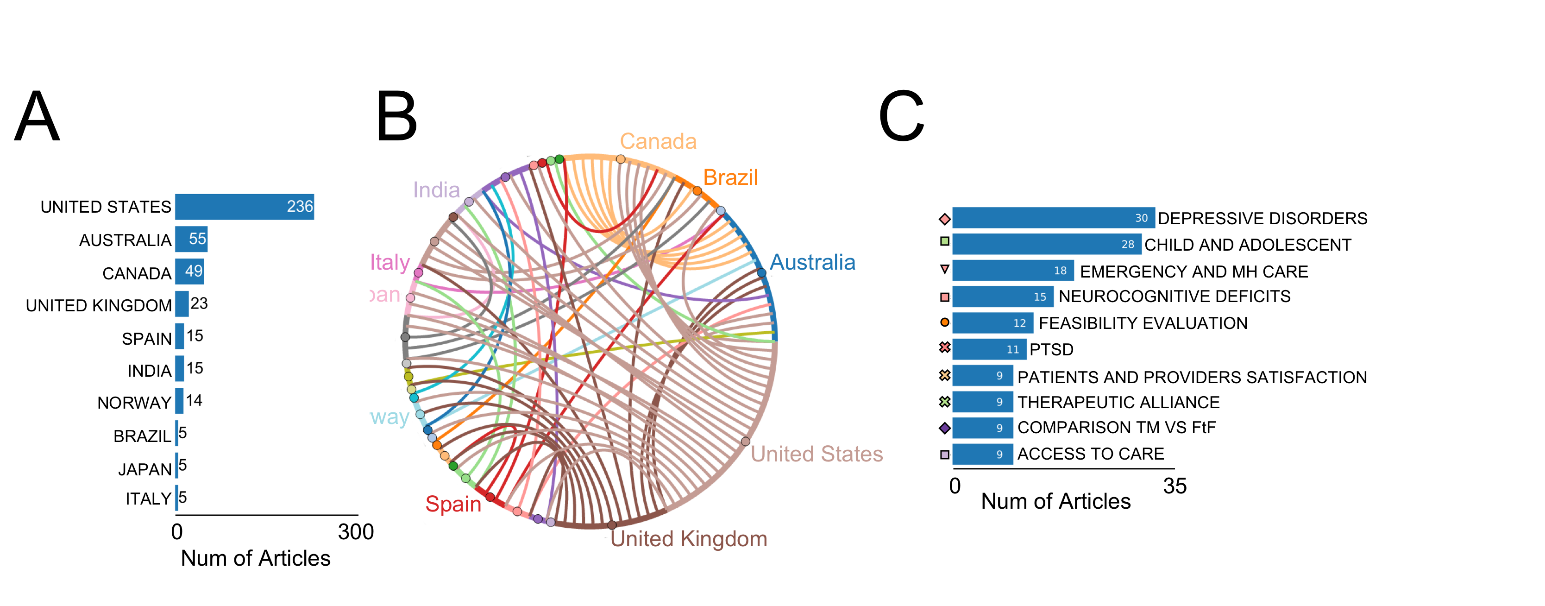


**A.** Number of articles contributed by each country to the whole dataset (top 10 countries). **B.** Chord diagram plotting international collaborations, where countries are arranged along the circumference, and are connected by arcs in case they share co-authorships. **C.** Top 10 topics and relative numeric representation in the dataset.

#### Figure S2. Italian providers’ responses to the survey on the use of tele-mental health during the COVID-19 pandemic.


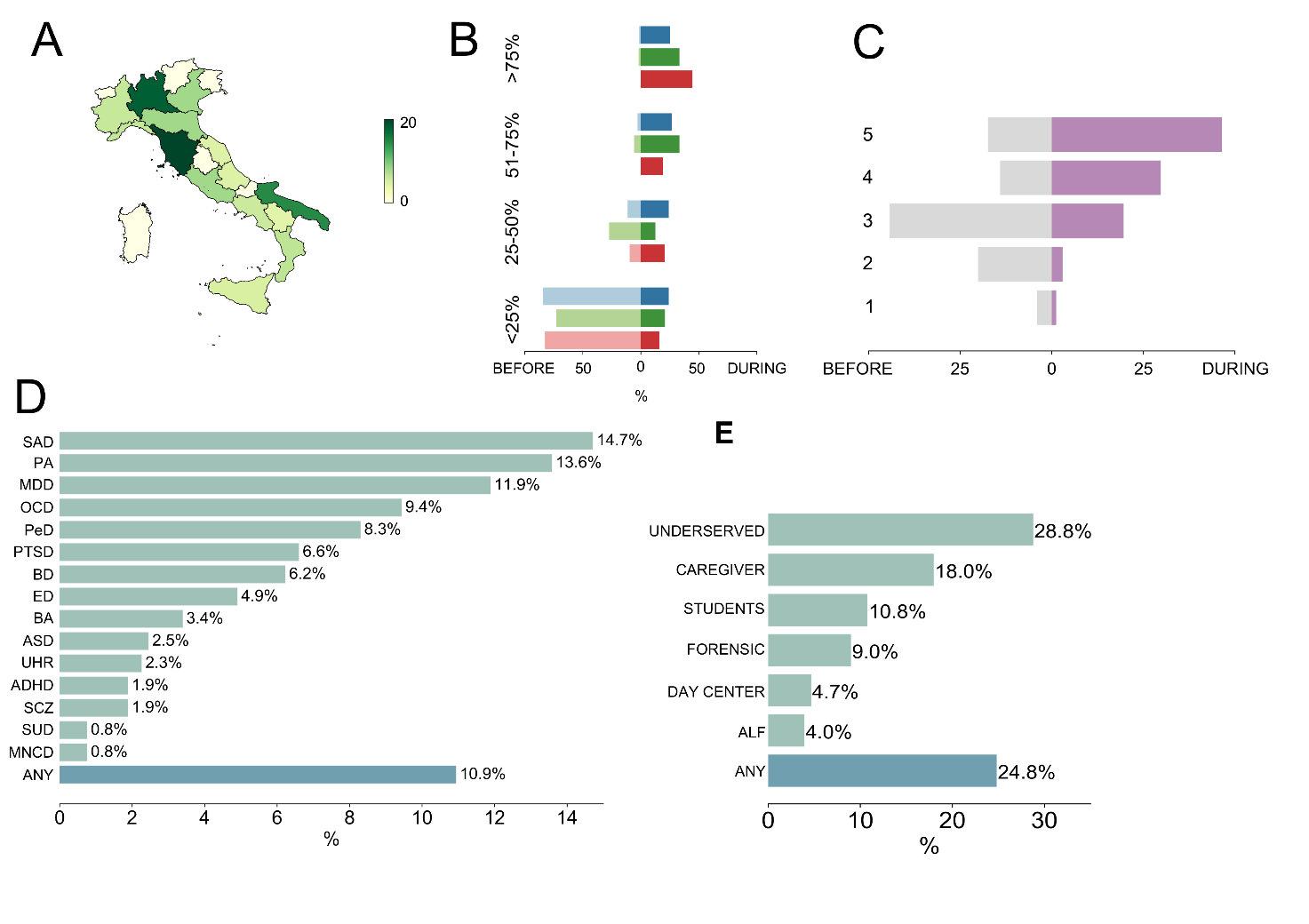


**A.** Breakdown of number of responses by region. **B.** Number of physicians (green), psychologists (red) and other mental health professionals (blue) offering tele-mental health for: more than 75%; 50-75%; 25-50%; less than 25% of their services, during and prior to the pandemic. **C.** Perceived usefulness of tele-mental health on a scale from 1 to 5 during and prior to the pandemic; **D.** Ranking of mental disorder diagnoses amenable to tele-mental care (ADHD, attention-deficit hyperactivity disorder; ASD, autism-spectrum disorders; BA, behavioral addiction; BD, bipolar disorders; ED, eating disorders; MDD, major depressive disorders; MNCD, major neurocognitive disorders; OCD, obsessive compulsive disorder; PA, panic disorder; PeD, personality disorders; PTSD, post-traumatic stress disorder; SAD, social anxiety disorder; SCZ, schizophrenia-spectrum disorders; SUD, substance use disorders; UHR, ultra-high risk for psychosis). **E.** Ranking of population groups amenable to tele-mental care (ALF, assisted living facility).

### Results S1. Data from the International survey on tele-mental health use during the COVID-19 pandemic

The survey was completed by 120 mental health care providers from several Countries. The highest number of responses came from the European Union (n=38), followed by Brazil (n=23), United Kingdom (n=9) and United States of America (n=9). Breakdown by Countries is depicted in Fig S3.a. Participants were evenly distributed by gender (48,3% female; 50,8% male). One third of respondents (n=44, 33,3%) were in the age range 31-40; age ranges 41-50, 51-60, and >60 were equally represented (n=22, 18,3%; n=21, 17,5%; n=26, 21,7%, respectively). Eleven respondents (9,2%) were aged 20-30 years. Fifty-three percent of respondents (n=64) were employed in the public sector, while 56 (47%) worked in the private sector. The sample included 72 (60%) physicians, 46 psychologists (38%) and only 2 other mental health workers. Half of respondents (n=60) reported that their area was provided with an electronic health record (EHR); 39 (33%) replied that EHR was not available in their area, and 21 (18%) did not know.

We observed a very high rate of disruption due to COVID-19, globally: 83% of the sample reported some level of disruption in their normal service provision. The most frequent reasons were the lockdown measures implemented by most Countries (n=70, 58%) and the reduction or block in non-urgent services (n=26, 22%). Three respondents reported that their ward had been converted to a COVID-clinic, and there was only one case of infection in our sample. Consistently with the results of the Italian survey, on a scale from 1 to 10, median of COVID-19-related disruption was 7 (IQR=5-8) (Fig S3.b).

A dramatic shift toward the use of tele-mental interventions could be observed in our sample. Nearly the total of our sample (n=109, 92%) reported using tele-mental care during the pandemic, but only 45% reported using tele-mental care prior to the COVID-19 crisis. Of note, 73% respondents reported using tele-mental care mostly or exclusively after the onset of the COVID-19 crisis (vs 4% prior to the pandemic) (Fig S3.c). The most prominent shift was observed among psychologists, as compared to physicians (91% vs 61%, Χ^2^=17,85, df=3, p<0,001) (Fig S3.c). Our data indicate that there were no difficulties in the transition to tele-mental health in respondents of > 50 years of age (Fig S4). Respondents provided a variable amount of care provisions through tele-mental care, ranging from less than 25% to more than 75%, with an even distribution. On the contrary, prior to the pandemic, tele-mental health was used for less than 25% of care provisions by 92% of respondents (n=91, over 99 valid responses) (Fig S3.d). In particular, we observed an increase in the use of video-teleconferencing (Fig S3.e). Usefulness perception improved robustly: 88% found tele-mental health much or very much useful during the pandemic, relative to 44% prior to the pandemic (Fig S3.f). Also internationally, most providers (39%, n=45 over 116 valid responses) used personal telecommunications at their own initiative, since less than one third of work settings were adequately equipped (26% and 20% in public and private work settings, respectively). Among those working in public settings, half reported that their employer introduced and enabled tele-mental health during the pandemic. Fig S3.g and Fig S3.h represent the rankings of diagnoses and population groups preferentially offered tele-mental care.

Similar to the responses in the Italian sample, only 29% of respondents thought that tele-mental health was as valid, accurate and effective as face-to-face care; 56% was not positive about the ability to establish a good doctor-patient relationship. However, as compared to Italian providers, a higher proportion of respondents felt that: tele-mental health could reduce the barrier of stigma, 58%; they were somewhat or very much prepared to use tele-mental health, 76%; they were satisfied with the care they are able to provide through tele-mental health, 67% (Fig S3.i).

#### Figure S3. International providers’ responses to the survey on the use of tele-mental health during the COVID-19 pandemic.

**A.** Breakdown of number of responses by country. **B.** COVID-19-related disruption in mental health service provision. **C.** Number of physicians and psychologists offering services by: exclusively face-to-face, mostly face-to-face, mostly tele-mental health, exclusively tele-mental, during and prior to the pandemic. **D.** Number of physicians and psychologists offering tele-mental health for: more than 75%; 50-75%; 25-50%; less than 25% of their services, during and prior to the pandemic. **E.** Tele-mental tools used during and prior to the pandemic (EHR, electronic health record; IM, instant messaging; STM, supported telemedicine systems; VTC, video-teleconferencing). **F.** Perceived usefulness of tele-mental health on a scale from 1 to 5 during and prior to the pandemic; **G.** Ranking of mental disorder diagnoses amenable to tele-mental care (ADHD, attention-deficit hyperactivity disorder; ASD, autism-spectrum disorders; BA, behavioral addiction; BD, bipolar disorders; ED, eating disorders; MDD, major depressive disorders; MNCD, major neurocognitive disorders; OCD, obsessive compulsive disorder; PA, panic disorder; PeD, personality disorders; PTSD, post-traumatic stress disorder; SAD, social anxiety disorder; SCZ, schizophrenia-spectrum disorders; SUD, substance use disorders; UHR, ultra-high risk for psychosis). **H.** Ranking of population groups amenable to tele-mental care (ALF, assisted living facility). **I.** Providers’ attitude towards tele-mental health.


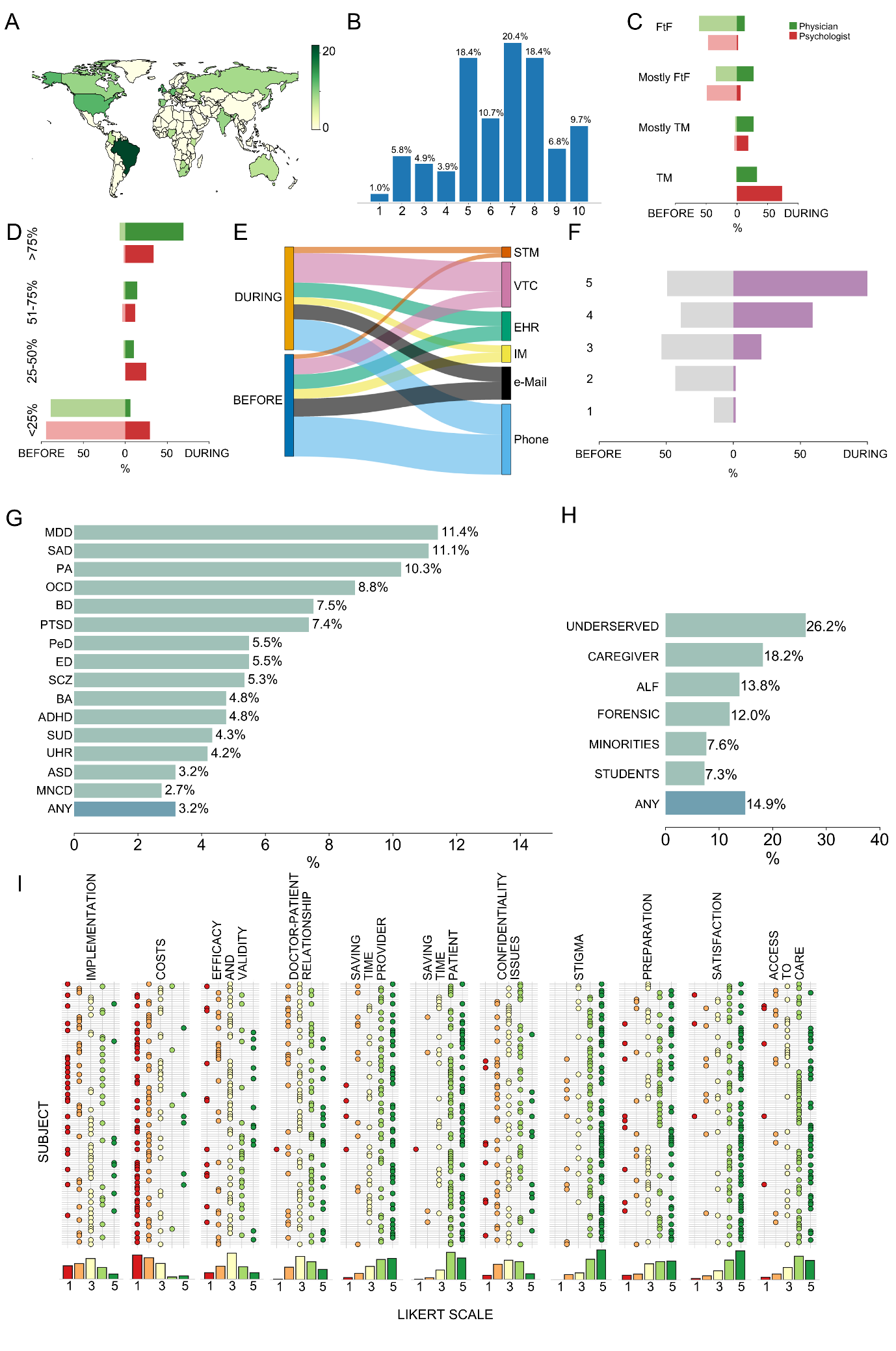


#### Figure S4. International providers’ responses: use of tele-mental health during the COVID-19 pandemic across age groups.


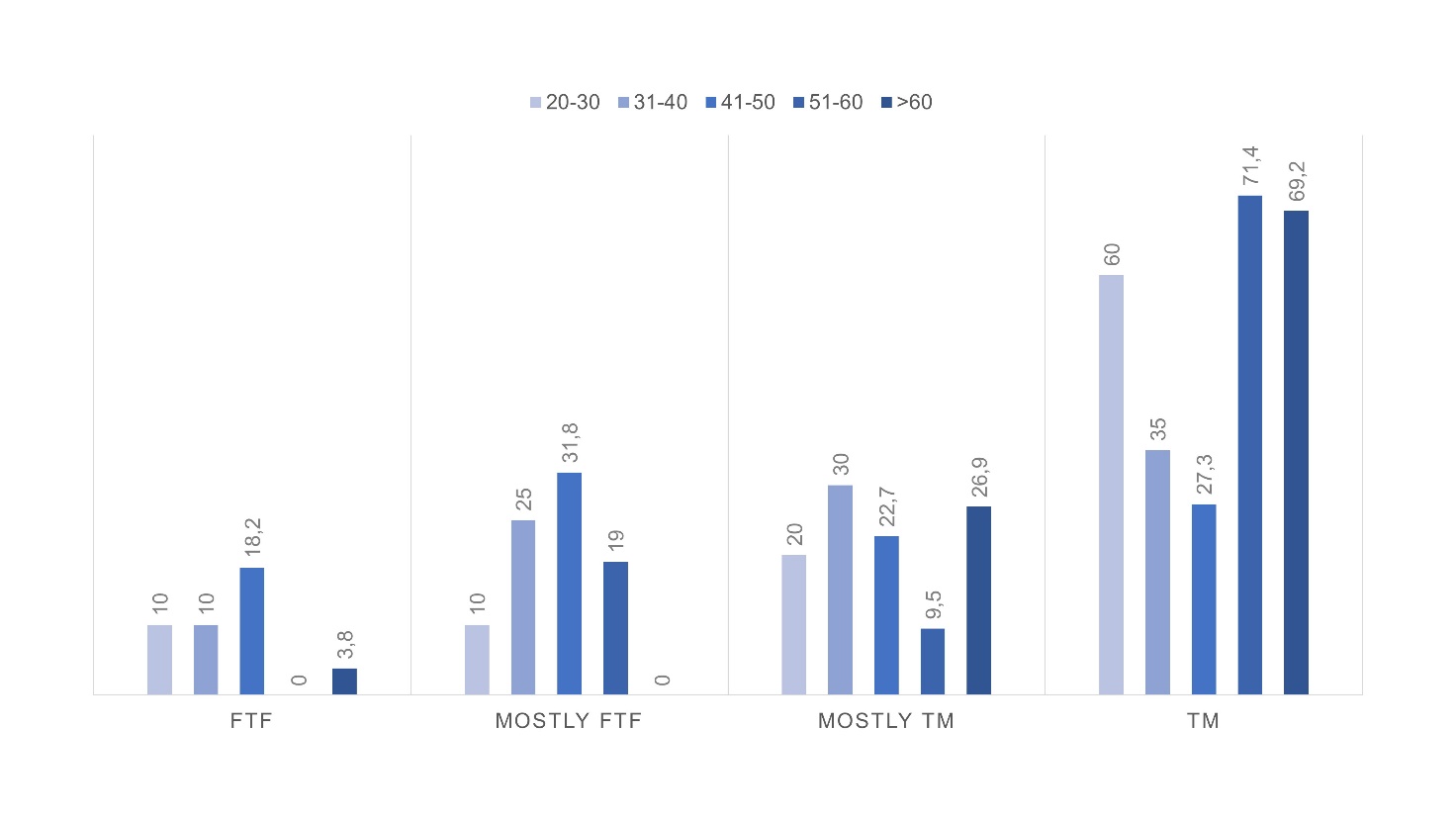


FtF, face-to-face; TM, tele-mental health

####
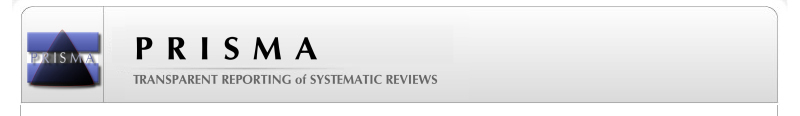
Figure S5. PRISMA 2009 Flow Diagram

**41** Eligible articles

**12** Eligible not included in meta-analysis as failing to report usable data

**Included**

**Eligibility**

**Screening**

**Identification**

**206** Excluded after full-text screening:

**12** inappropriate control group (waiting list or mixed TM/FtF)

**136** not comparing TM to FtF

**34** not reporting on patients’ satisfaction

**4** not in mental health

**13** system development/ description/ evaluation

**3** educational use

**4** overlapping datasets

From: Moher D, Liberati A, Tetzlaff J, Altman DG, The PRISMA Group (2009). Preferred Reporting Items for Systematic Reviews and Meta-Analyses: The PRISMA Statement. PLoS Med 6(6): e1000097. Doi: 10.1371/journal.pmed1000097

For more information, visit www.prisma-statement.org.

**632** Articles identified through database searching:

**292** Web of Knowledge^SM^

**340** Scopus®

**29** Articles included in meta-analysis:

**11** Telepsychiatry

**17** Telepsychology/Counselling

**1** Both

**148** Excluded on basis of abstract:

**78** Reviews

**37** Study protocols

**27** Case studies/series

**6** Not in English

**247** Full-text articles assessed for eligibility

**22** Excluded on basis of title

**395** Abstracts screened

**417** Articles after duplicates removed

**21** Additional articles identified through manual search

#### Table S4. Characteristics of the eligible studies not included in meta-analysis

|  | **Country** | **Diagnosis** | **Population** | **Study type** | **Intervention** | **Modality** | **Satisfaction measure** | **Sample size**  **(respondant/**  **randomized)** | **Main findings** |
| --- | --- | --- | --- | --- | --- | --- | --- | --- | --- |
| Burton, et al. 2016 (13) | Multicentric: Romania, Spain and UK | MDD | Adult outpatients | RCT, Pilot | Interactive system with avatar “Help4Mood” | Virtual agent | Qualitative | TM: 11/13 (84,6%)  FtF: 9/14 (64,3%) | All participants would use and recommend Help4Mood |
| Cheng, et al. 2018 (14) | Hong Kong | Any mental disorder | Adult outpatients | Case-control, pilot | Telepsychiatry | video-teleconferencing | Custom | TM: 86  FtF: 249 | Favourable response to teleconsultation |
| Comer, et al. 2017 (15) | USA | Disruptive disorder | Children 3-5 y.o. and caregiver(s) | RCT | Parent-child interaction therapy | video-teleconferencing | CSQ-8 | TM: 18/20 (90%)  FtF: 17/20 (85%) | mean TM: 30,1 mean FtF: 28,5  (max: 32) |
| Crowe, et al. 2016 (16) | USA | Any mental disorder | Deaf adult outpatients | Case-control | Telepsychiatry | video-teleconferencing | Patient satisfaction of services | TM: 13  FtF: 11 | 100% satisfaction with TM; 81,82% satisfaction with FtF |
| Iiboshi, et al. 2020 (17) | Japan | Neurocognitive disorders | Elderly patients | Comparative, crossover | Montreal Cognitive Assessment Tool | video-teleconferencing | Custom | TM: 39  FtF: 44 | High level of overall satisfaction (mean±SD 5,0±1,1) on a scale from 1 [VTC is much worse than FtF] to 7 [VTC is much better than FtF] |
| Jones, et al. 2012 (18) | USA | Any mental disorder | Adult military | Comparative | Mental screening after deployment | video-teleconferencing | Custom | TM: ns  FtF: ns | Preference for FtF screening |
| Khasanshina, et al. 2008 (19) | USA | Any mental disorder | College students | Comparative | Counselling | video-teleconferencing | Custom | TM: 22/53 (41,5%)  FtF: 495 | Clients rated TM as a valuable resource |
| Modai, et al. 2006 (20) | Israel | Any mental disorder | Adult outpatients | Comparative | Telepsychiatry | video-teleconferencing | Patient satisfaction questionnaire | TM: 39/49 (79,6%)  FtF: 42 | Patients were generally satisfied |
| Nelson, et al. 2003 (21) | USA | MDD | Children 8-14 y.o. and caregiver(s) | RCT | CBT | video-teleconferencing | Telemedicine satisfaction questionnaire | TM: 14/19 (73,7%)  FtF: 14/19 (73,7%) | All participants satisfied with TM; most preferred TM over FtF; most common concern not being able to hear well over the video |
| Rohland, et al. 2001 (22) | USA, *underserved* | Any mental disorder | Adult outpatients | Comparative, crossover | Telepsychiatry | video-teleconferencing | Satisfaction with ambulatory services 4.0 | TM: ns  FtF: ns | TM > FtF in convenience, ease, technical skills, attention given and time spent; FtF > VTC in self-reported outcome, helpfulness, eye contact, and overall satisfaction |
| Urness, et al. 2006 (23) | Canada | Any mental disorder | Adult outpatients | Comparative | Telepsychiatry | video-teleconferencing | Client satisfaction survey | TM: 39  FtF: 20 | Comments generally positive; lower satisfaction in TM group as compared to FtF. 96%, satisfaction with overall outcome; 100%, satisfied that doctor listerned to them; 78%, satisfied with support and encouragement; 85%, satisfaction with perceived ability to talk; 92%, able to present same information as in FtF setting |
| Ziemba, et al. 2014 (24) | USA, *underserved* | PTSD | Adult military | RCT, equivalence | CBT | video-teleconferencing | Patient satisfaction survey | TM: 7/9 (77,8%)  FtF: 6/9 (66,7%) | mean TM: 98,1; mean FtF: 92,1  (max 100) |

CBT, cognitive behavioral therapy; CSQ-8, Client Satisfaction Questionnaire; FtF, face-to-face; MDD, Major depressive disorder; ns, not stated; PTSD, Post-traumatic stress disorder; RCT, randomized controlled trial; TM, telemental; y.o., years old.

#### Figure S6. Forest plot showing the overall effect size for the comparison of satisfaction scores between tele-mental and face-to-face interventions for mental disorders. Positive values favor tele-mental, while negative values favor face-to-face.


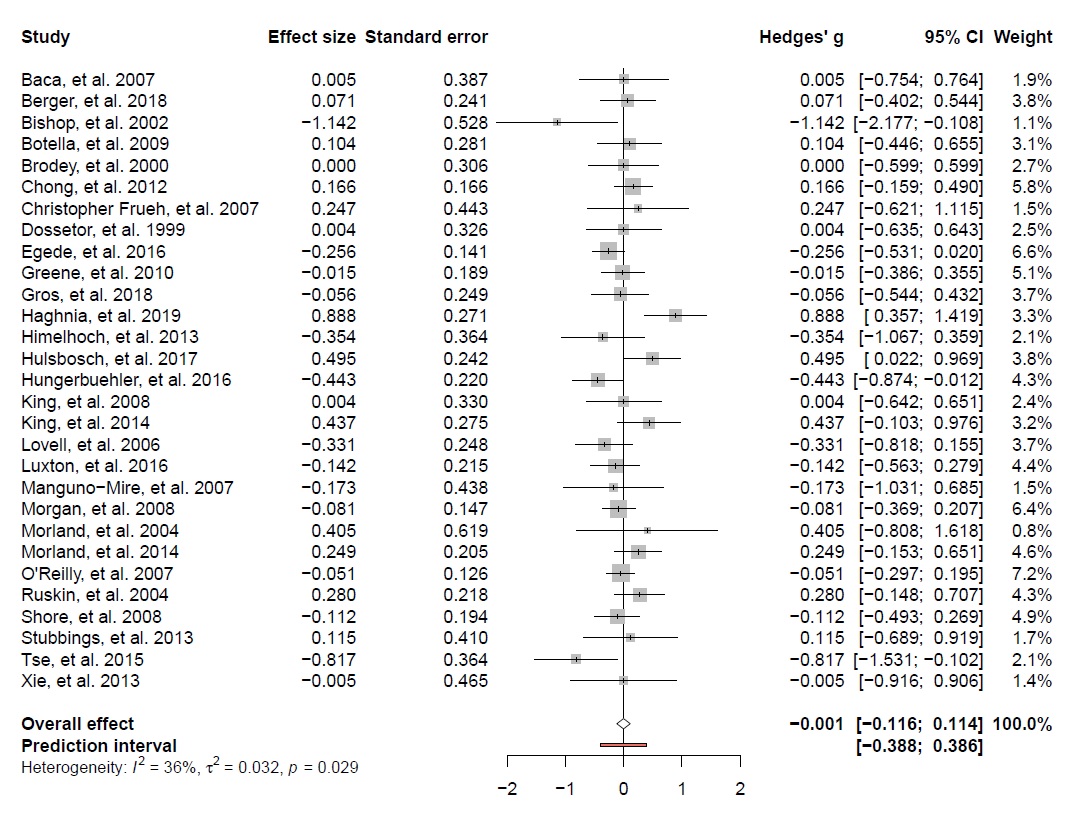


#### Figure S7. Influence analysis with the Graphic Display of Heterogeneity (GOSH) plot


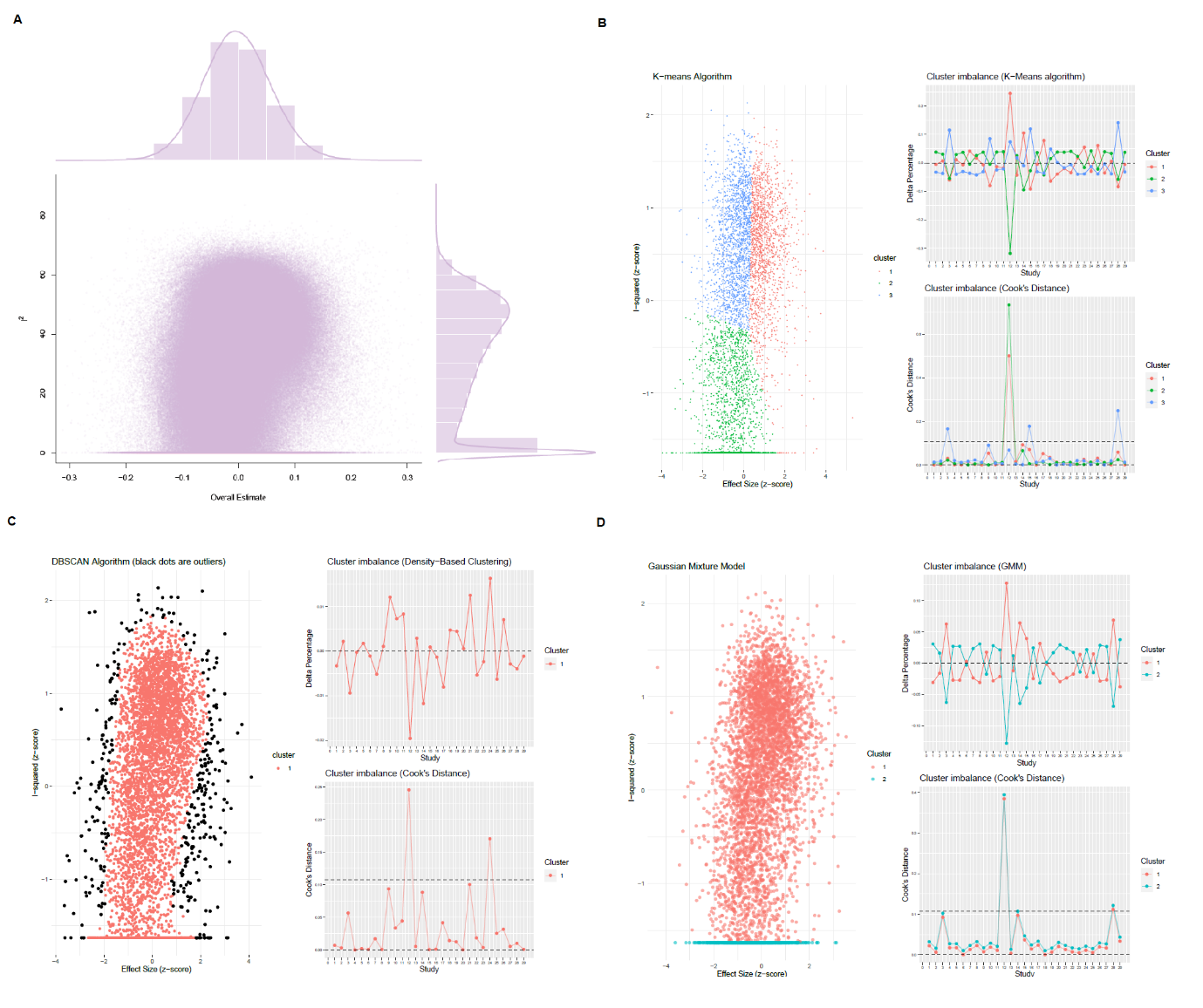


**A.** GOSH plot showing the meta-analysis models fitted to all 2^k-1^ possible combinations of the included studies (x-axis, pooled effect size; y-axis, between-study heterogeneity). **B.** k-means algorithm. **C.** DBSCAN. **D.** Gaussian Mixture Model. The three clustering (also known as supervised machine learning) algorithms are implemented in the *gosh.diagnostics* function of the R *dmetar* package. They detected study 12 (Haghnia, et al. 2019) as the study mostly contributing to the cluster imbalance.

#### Figure S8. Graphic Display of Heterogeneity (GOSH) plot, showing effect size-heterogeneity patterns.


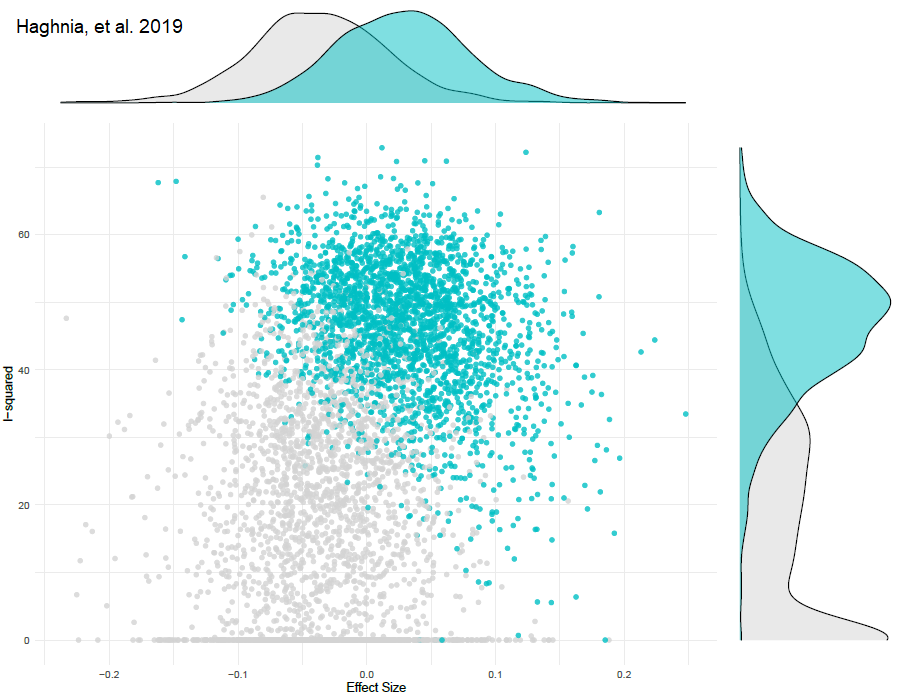


Meta-analysis models of all *2^k-1^* possible study combinations including or excluding Haghnia, et al. 2019 [(48)](https://paperpile.com/c/69gCR7/Cb9q) are depicted in green and gray, respectively. Haghnia, et al. 2019 contributes to cluster imbalance, shifting models toward higher heterogeneity estimates, and slightly more positive effect sizes.

### Results S2. Supplementary influence diagnostics

The results of supplementary influence diagnostics corroborated the findings of the influence analysis performed according to the GOSH plot method.

The 95% CI of the overall effect size (Hedges’ g) for the comparison of satisfaction levels with tele-mental vs face-to-face interventions is comprised between g=-0,116 and g=0,114. Again, the only study whose 95% CI was not overlapping with the pooled effect’s CI was Haghnia, et al. 2019.

The sensitivity analysis with the Leave-One-Out method revealed that overall effect size could be influenced by Haghnia, et al. 2019, as well (as summarized in Fig S4).

We plotted the effect size recalculated each time omitting one study in two forest plots, ordered by heterogeneity (as measured by I^2^) and effect size, respectively (Fig S5). The lowest heterogeneity (I^2^=17%) was obtained by removing Haghnia, et al. 2019. When this study was removed, we observed a slight shift of the effect size toward negative values, that is favouring face-to-face over tele-mental interventions (Fig S6).

#### Figure S9. Influence analysis with the Leave-One-Out method


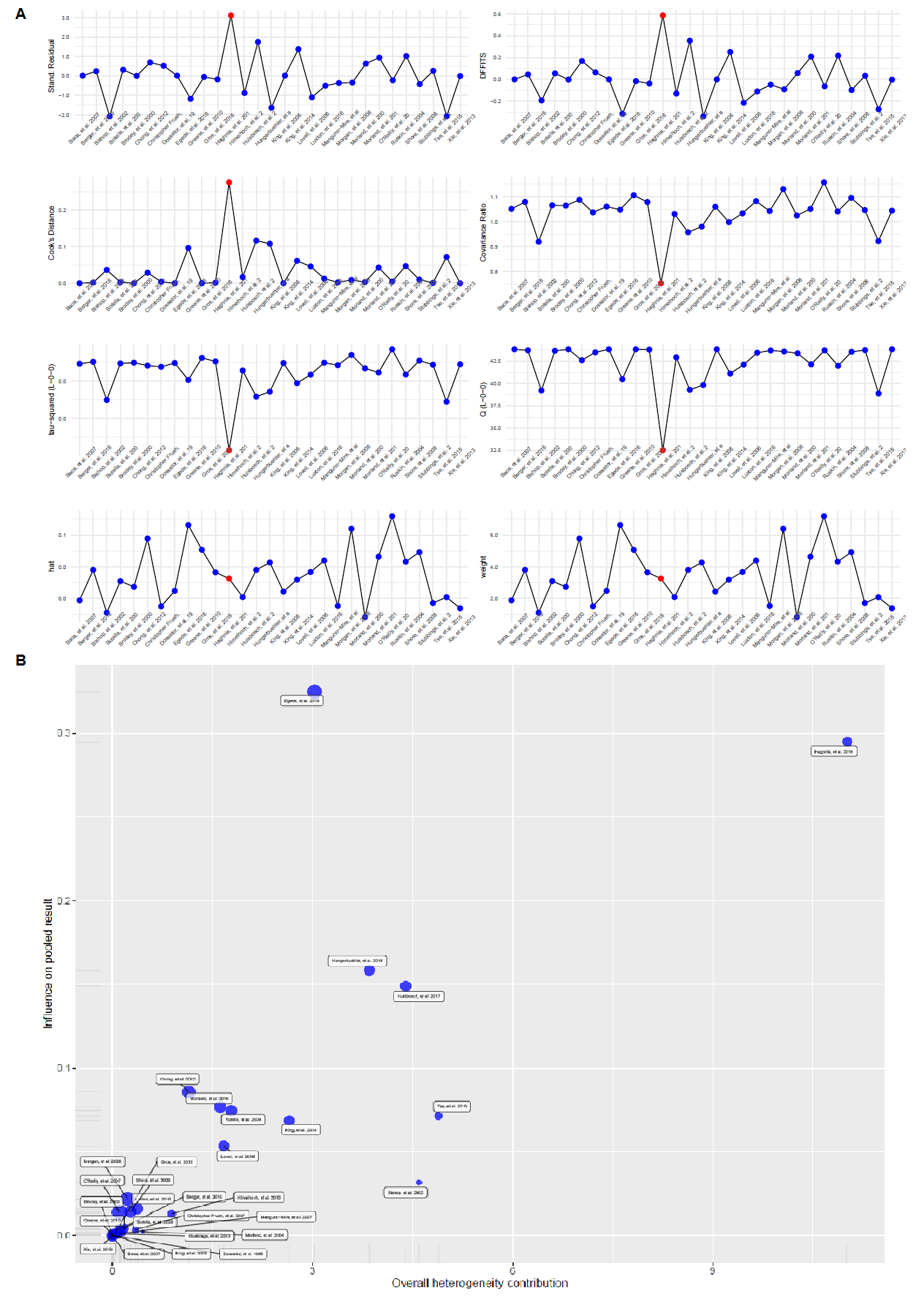


**A.** Parameters of the influence analysis: standardized residuals, dffits, Cook’s distance, covariance ratio, tau^2^, Q, hat, and weight. Haghnia, et al. 2019 is identified as an influential study according to the cut-offs proposed by Viechtbauer and Cheung (1) and marked with red dots. **B.** The Baujat plot shows each study contribution to overall heterogeneity (x-axis) and effect size (y-axis). Haghnia, et al. 2019 lies on the right upper corner of the plot, meaning it contributes substantially to both heterogeneity and overall effect size (medium weight).

#### Figure S10. Forest plots of the overall effect sizes recalculated with the Leave-One-Out method, ordered by heterogeneity (A) and effect size (B)


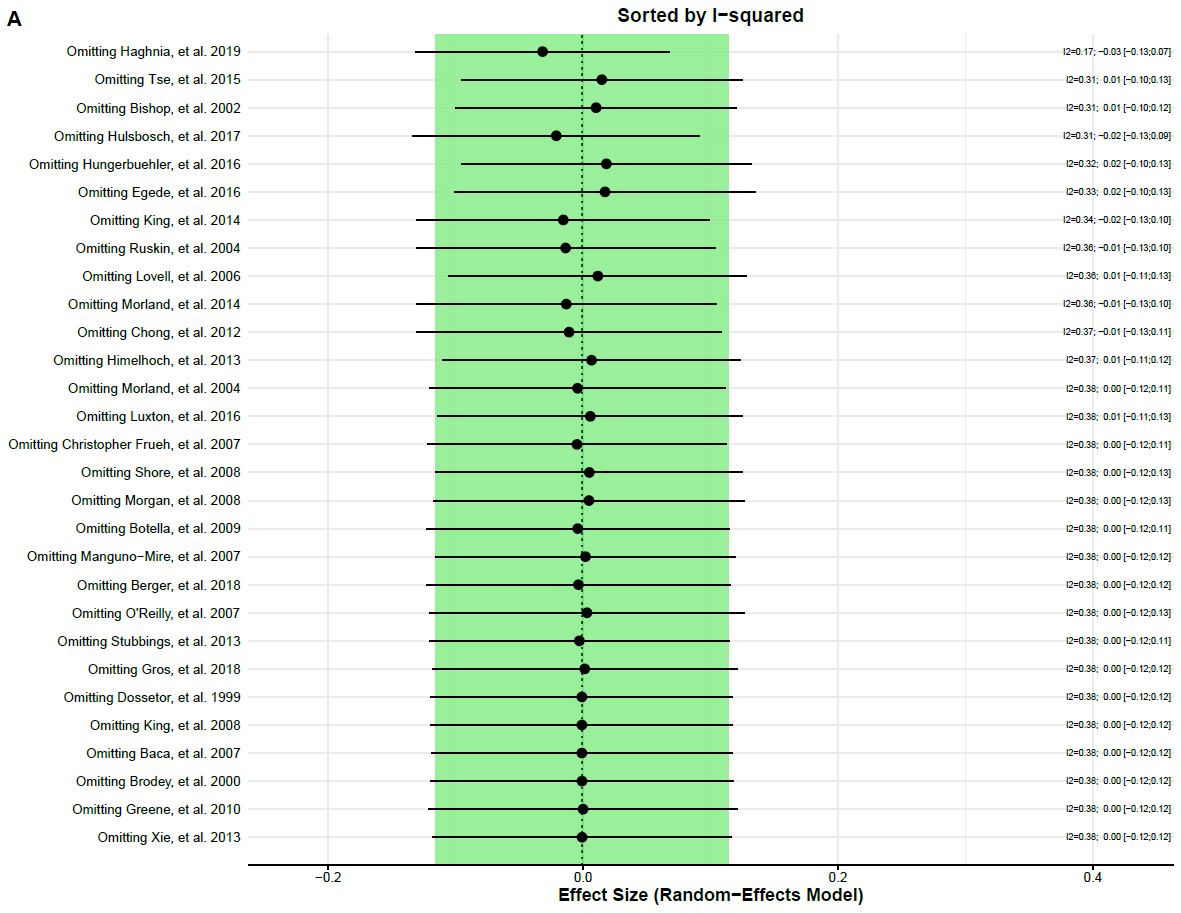


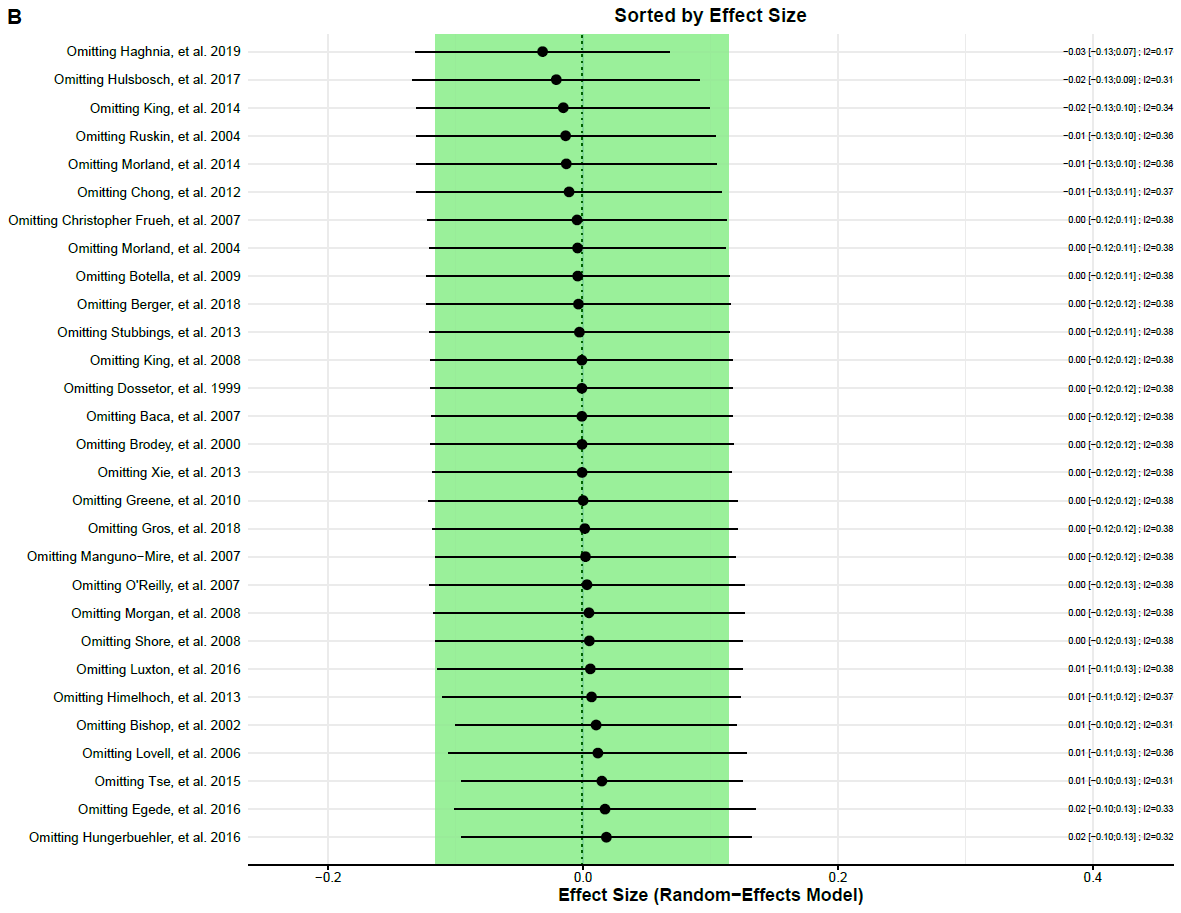


#### Figure S11. Forest plot after removal of the detected outlier (Haghnia, et al. 2019)


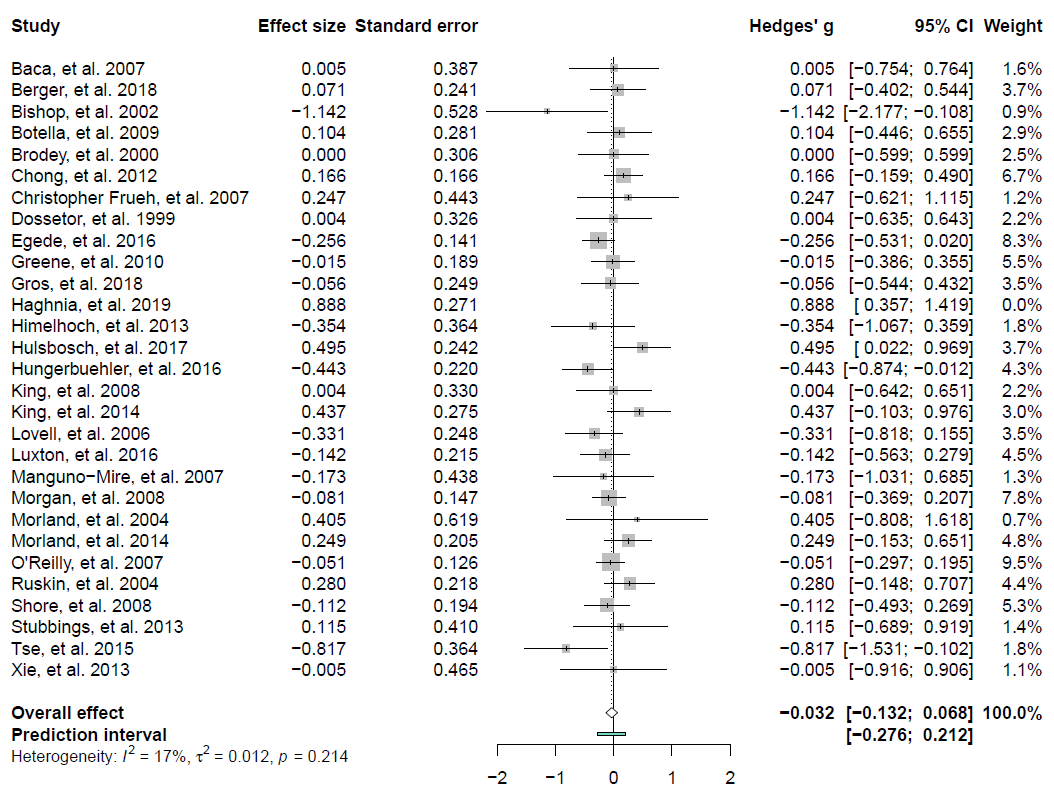


Overall effect size (Hedges’ g) for the comparison of satisfaction levels with tele-mental vs face-to-face interventions in patients with mental disorders, after removal of Haghnia, et al. 2019, which emerged from influence diagnostics to explain most of the observed between-study heterogeneity. Upon removal of the study, the I^2^ index dropped to 17% (low heterogeneity), and heterogeneity was no longer significant (Q=32,51, p=0,214). However, the impact on the overall effect size was negligible.

#### Figure S12. Subgroup analysis for mental disorder diagnosis


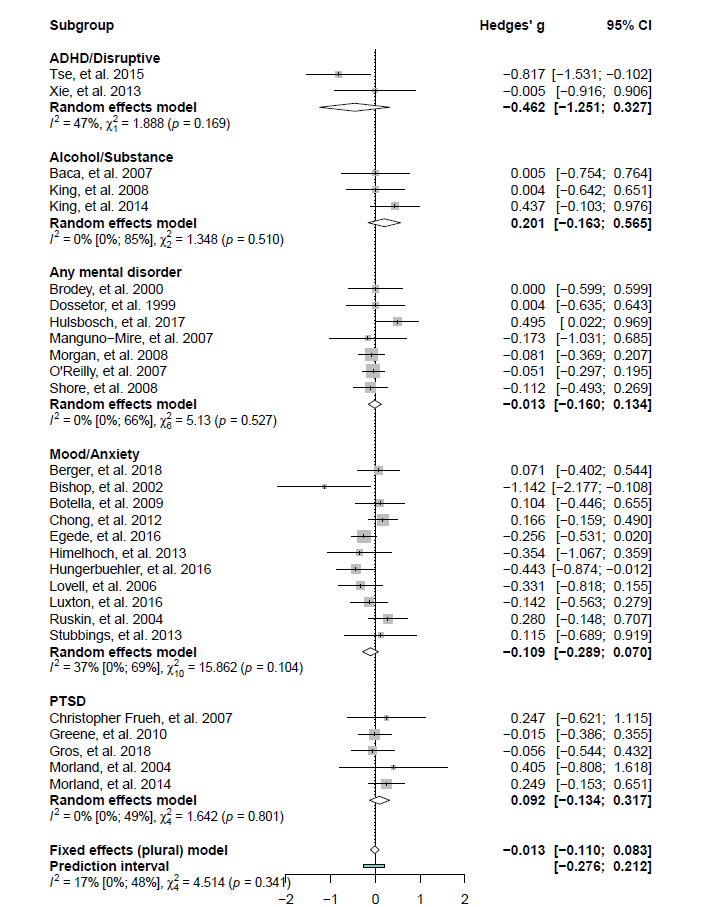


Although the pooled Hedges’ g of the subgroups vary from -0,46 (in favour of face-to-face) to 0,20 (in favour of tele-mental), the between-group heterogeneity was not statistically significant (p=0,34).

#### Figure S13. Subgroup analysis for population type


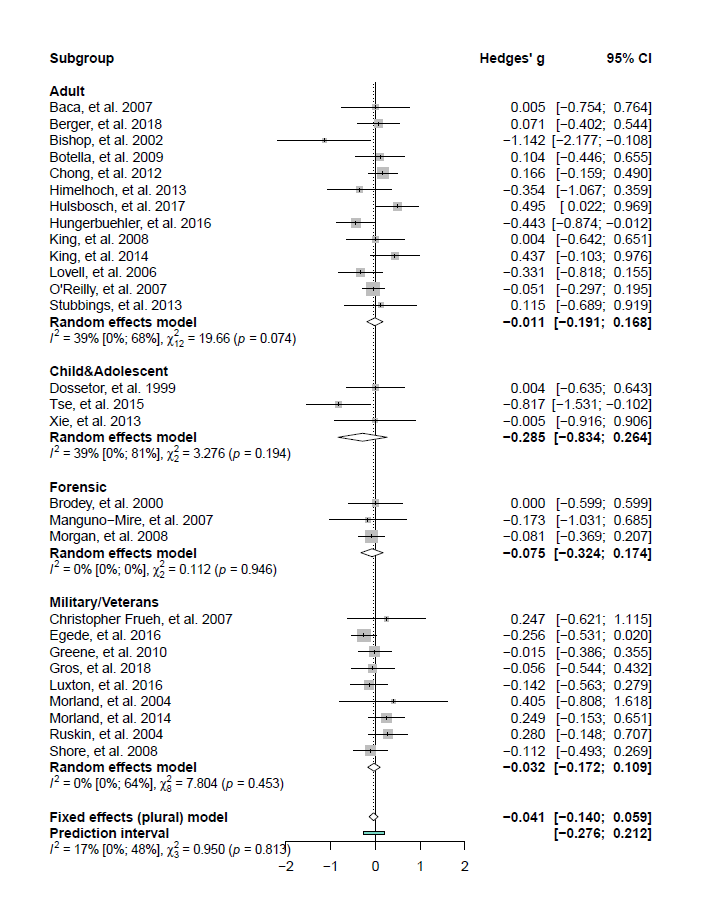


#### Figure S14. Subgroup analysis for served (no) vs underserved (yes) area or community


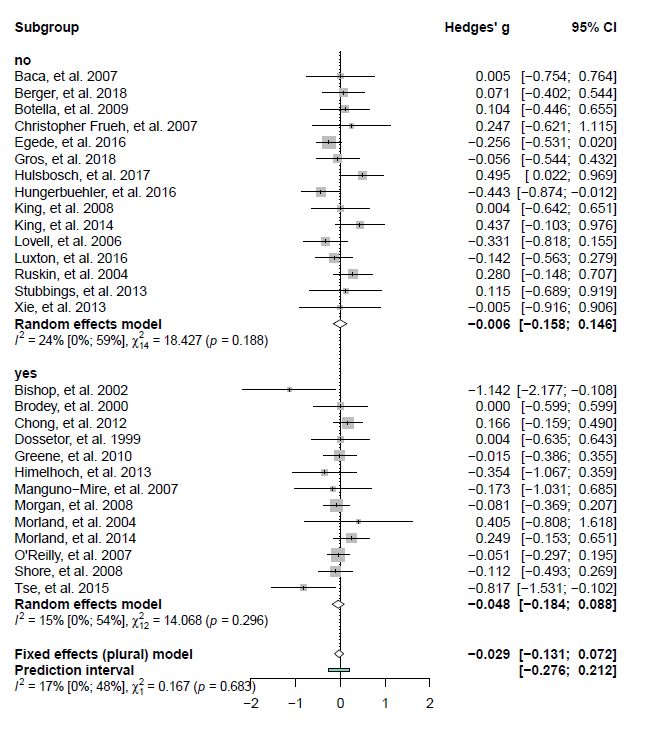


#### Figure S15. Subgroup analysis for non-RCT vs RCT study design


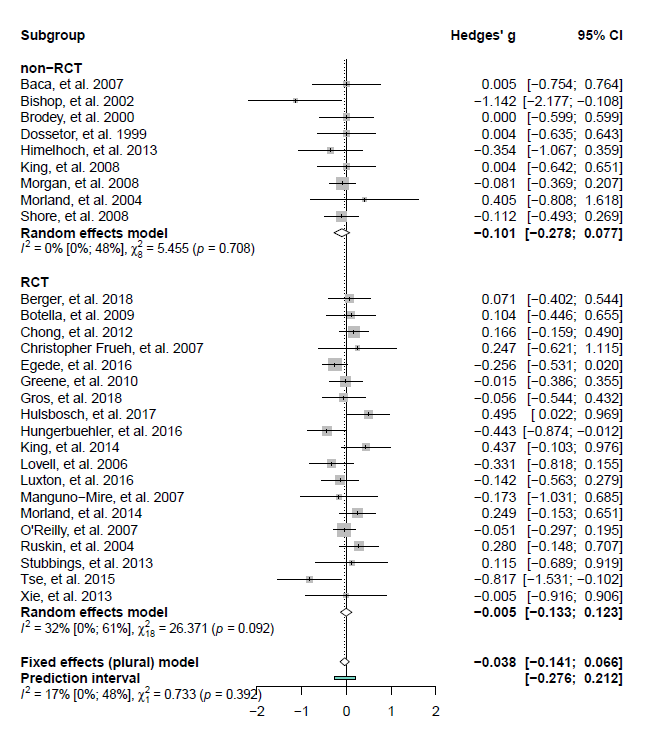


#### Figure S16. Subgroup analysis for custom vs standardized satisfaction scale


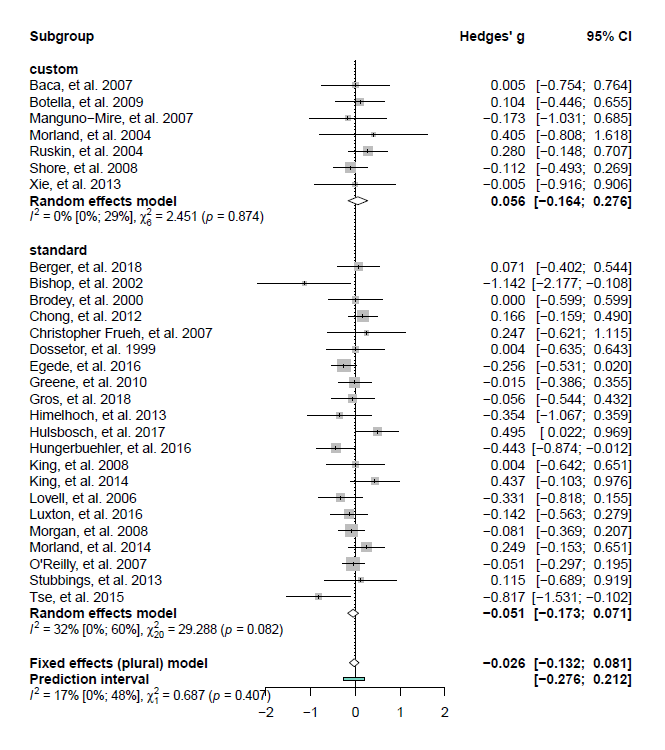


#### Figure S17. Meta-regression: Publication year


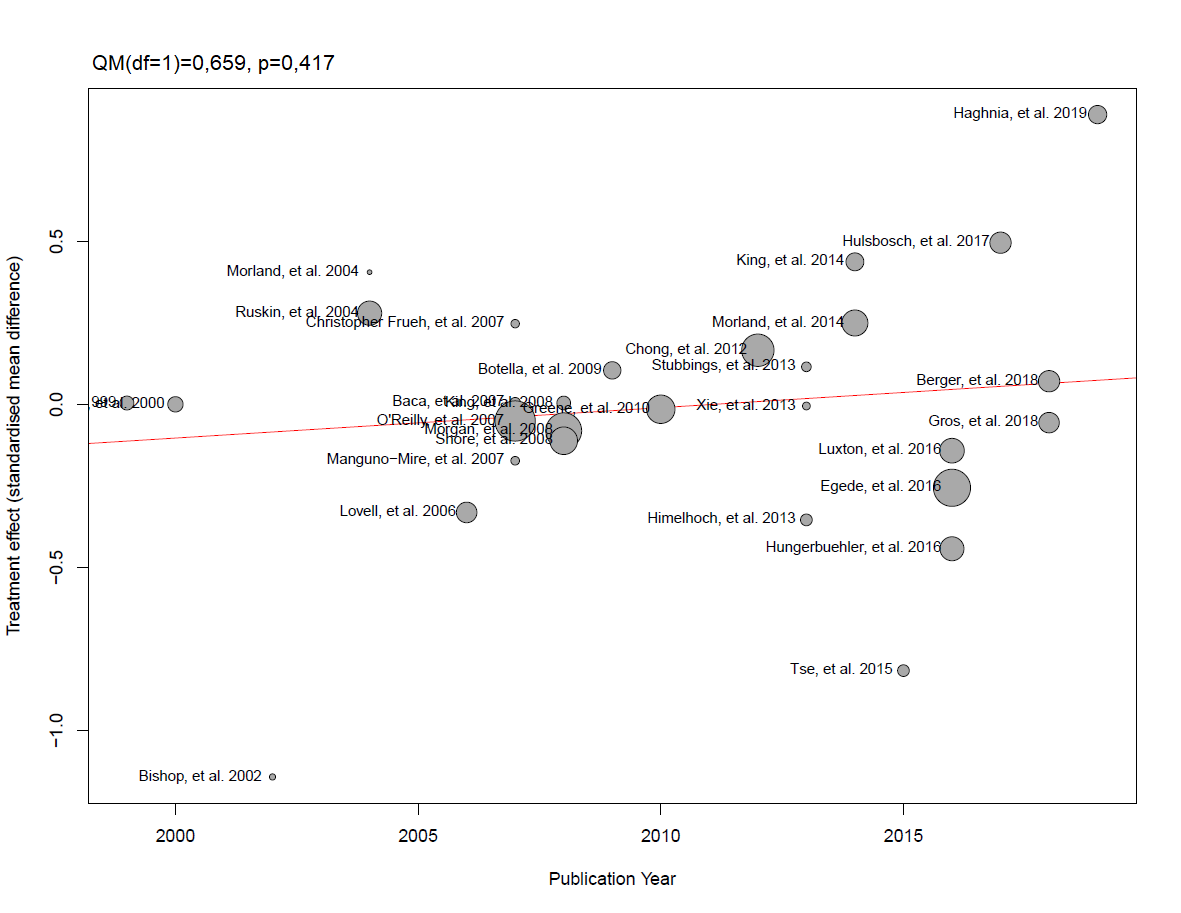


#### Figure S18. Meta-regression: Age


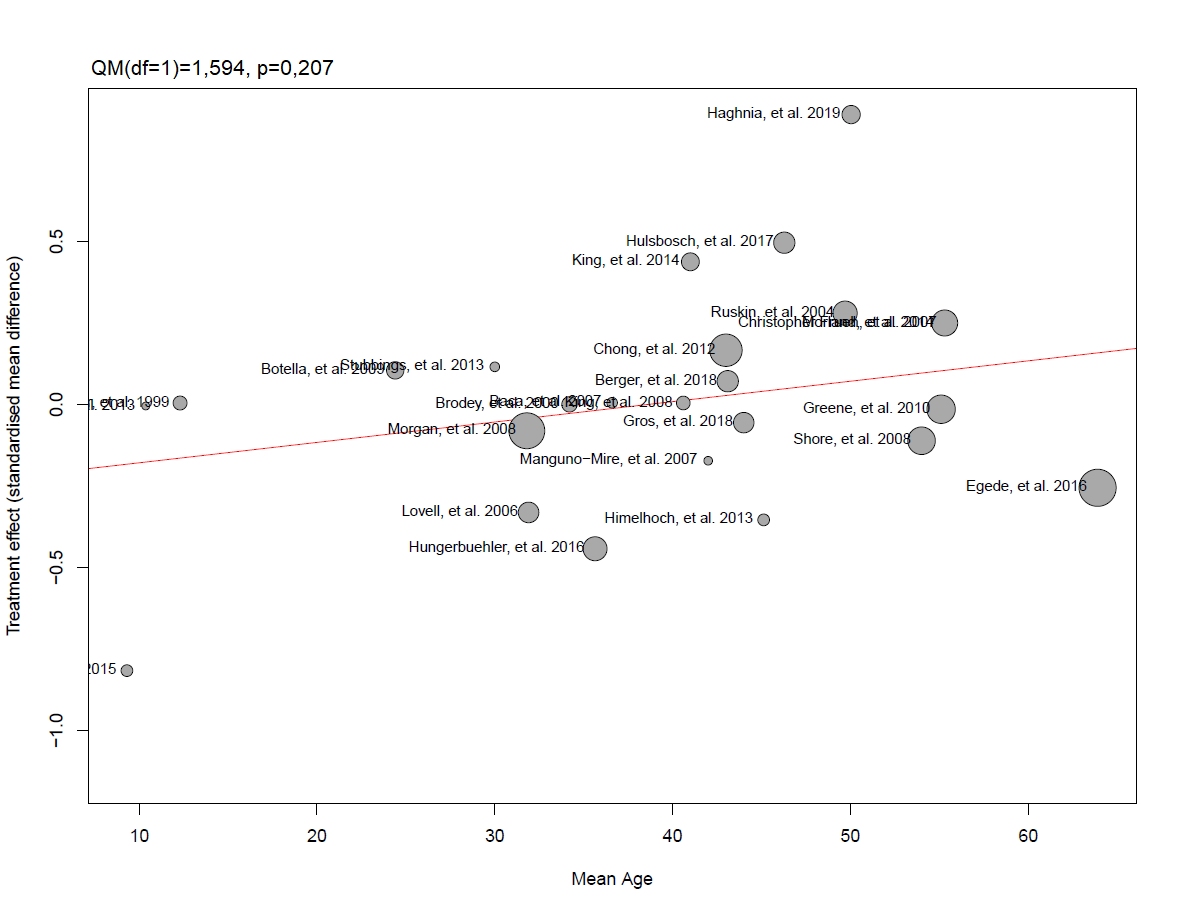


#### Figure S19. Meta-regression: Gender


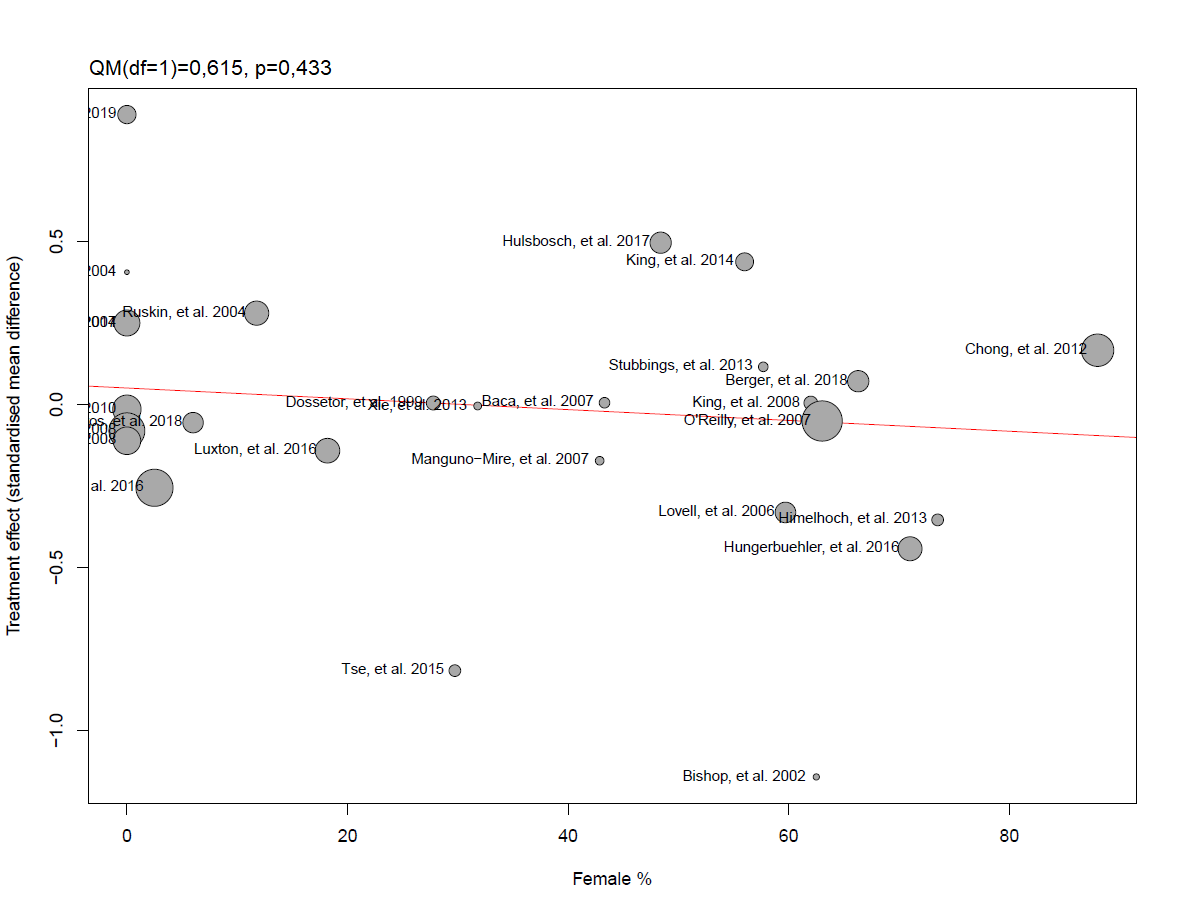


#### Figure S20. Meta-regression: Intervention duration


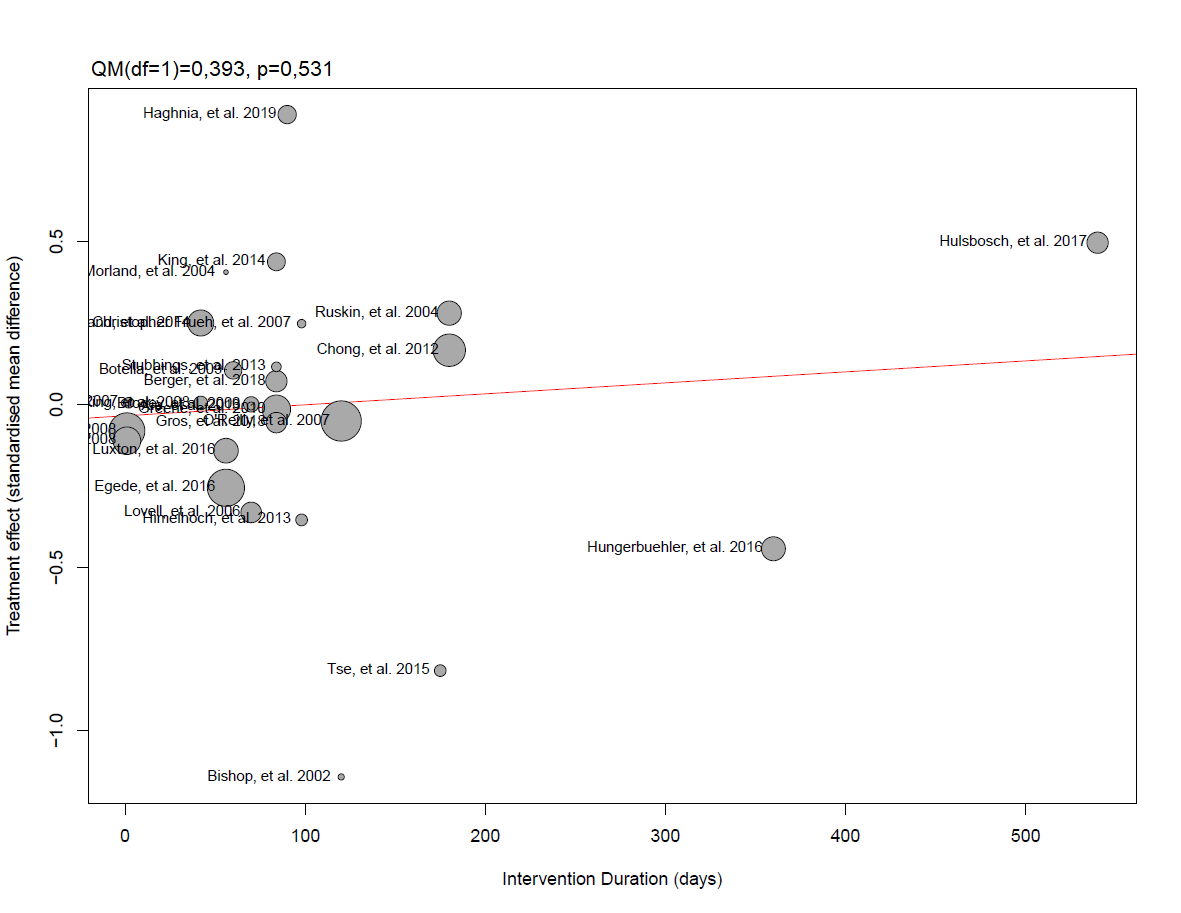


#### Figure S21. Meta-regression: Sample size


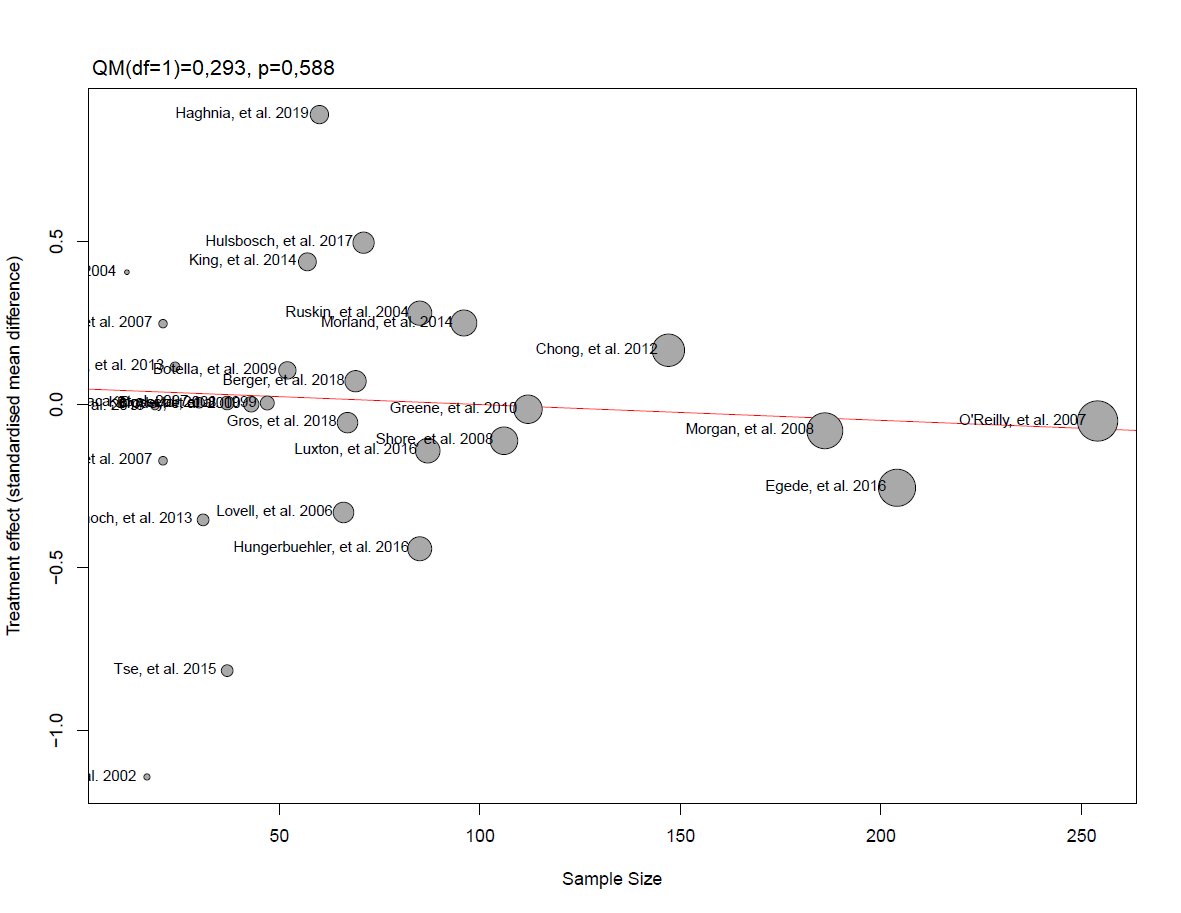


#### Figure S22. Assessment of small sample publication bias with the Funnel Plot


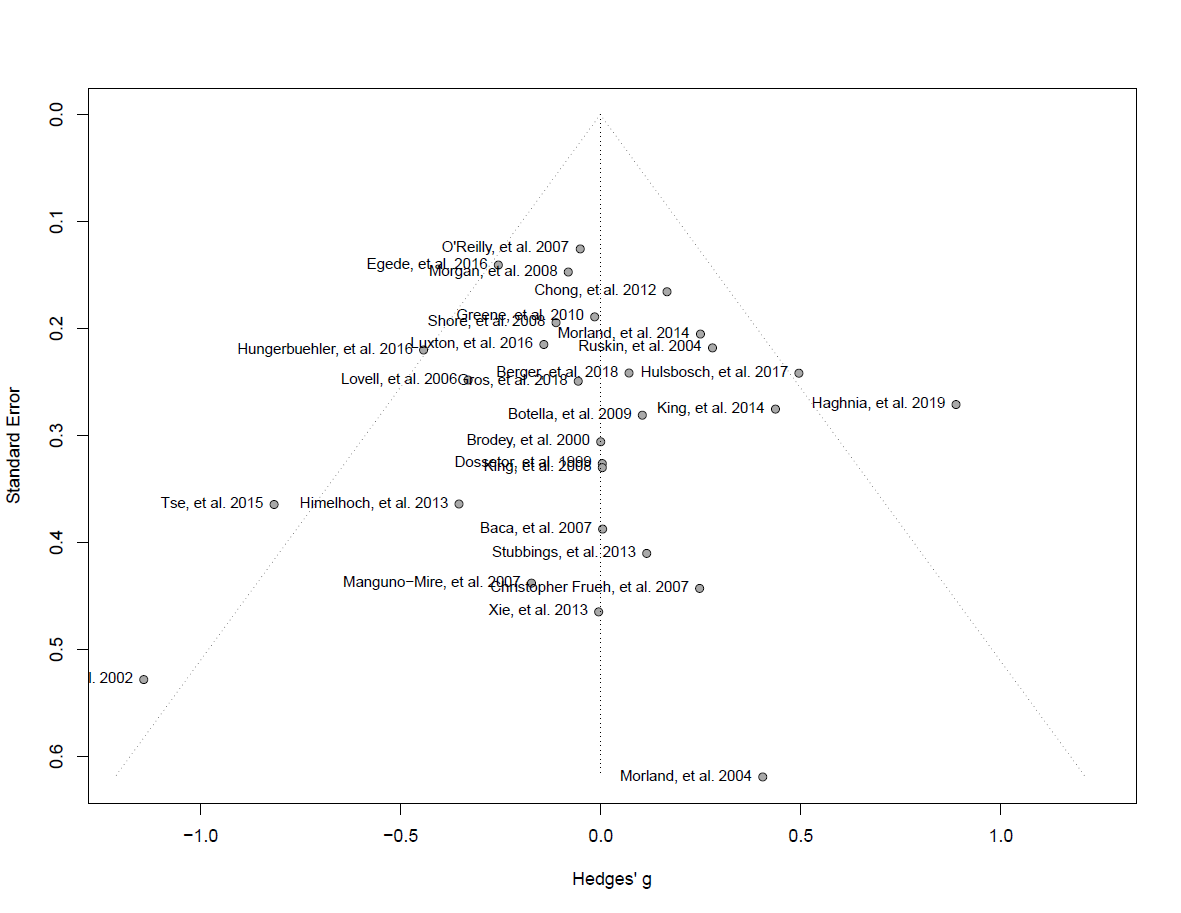


#### Figure S23. Risk of bias summary: Authors’ judgements about each risk of bias domain for each included study


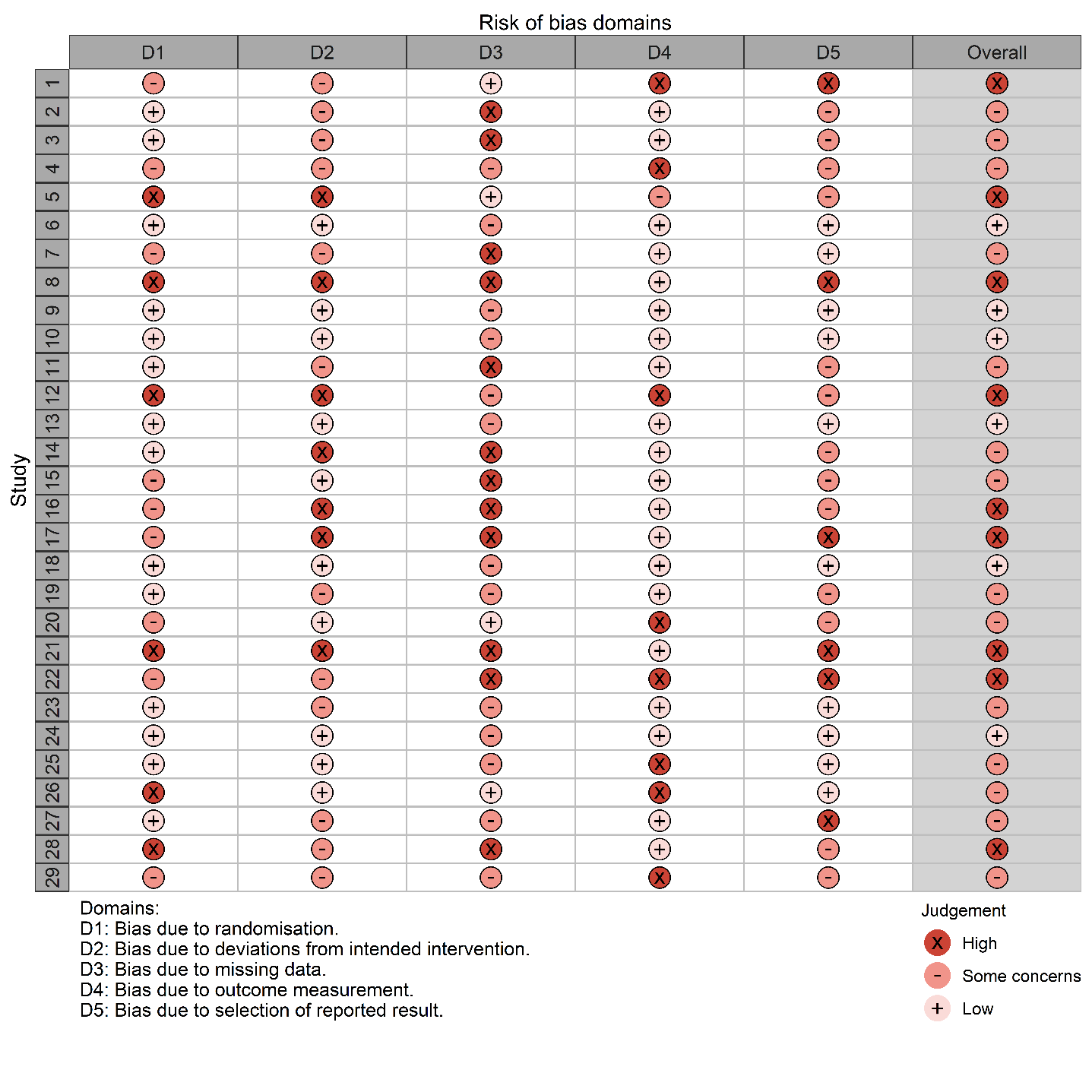


| **Studies:** | | |
| --- | --- | --- |
| 1: Baca, et al. 2007 | 11: Gros, et al. 2018 | 21: Morgan, et al. 2008 |
| 2: Berger, et al. 2018 | 12: Haghnia, et al. 2019 | 22: Morland, et al. 2004 |
| 3: Bishop, et al. 2002 | 13: Himelhoch, et al. 2013 | 23: Morland, et al. 2014 |
| 4: Botella, et al. 2009 | 14: Hulsbosch, et al. 2017 | 24: O’Reilly, et al. 2007 |
| 5: Brodey, et al. 2000 | 15: Hungerbuehler, et al. 2016 | 25: Ruskin, et al. 2004 |
| 6: Chong, et al. 2012 | 16: King, et al. 2008 | 26: Shore, et al. 2008 |
| 7: Christopher Frueh, et al. 2007 | 17: King, et al. 2014 | 27: Stubbings, et al. 2013 |
| 8: Dossetor, et al. 1999 | 18: Lovell, et al. 2006 | 28: Tse, et al. 2015 |
| 9: Egede, et al. 2016 | 19: Luxton, et al. 2016 | 29: Xie, et al. 2013 |
| 10: Greene, et al. 2010 | 20: Manguno-Mire, et al. 2007 |  |

#### Figure S24. Risk of bias summary: Authors’ judgements about each risk of bias domain for all included study


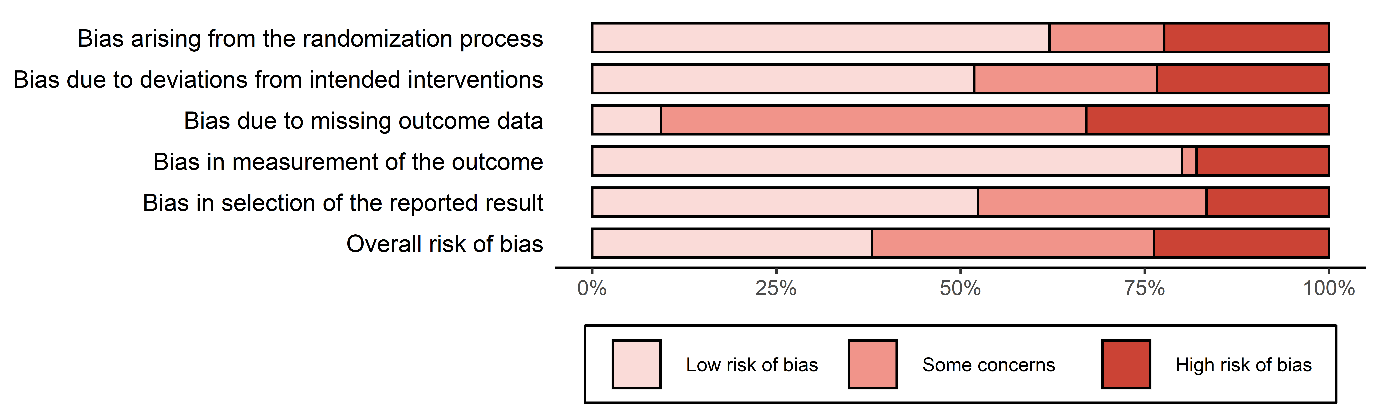


### References

1. Viechtbauer W, Cheung MW (2010): Outlier and influence diagnostics for meta-analysis. *Res Synth Methods*. 1:112-125.

2. Baujat B, Mahe C, Pignon JP, Hill C (2002): A graphical method for exploring heterogeneity in meta-analyses: application to a meta-analysis of 65 trials. *Stat Med*. 21:2641-2652.

3. Titov N, Andrews G, Davies M, McIntyre K, Robinson E, Solley K (2010): Internet treatment for depression: a randomized controlled trial comparing clinician vs. technician assistance. *PLoS One*. 5:e10939.

4. O'Reilly R, Bishop J, Maddox K, Hutchinson L, Fisman M, Takhar J (2007): Is telepsychiatry equivalent to face-to-face psychiatry? Results from a randomized controlled equivalence trial. *Psychiatr Serv*. 58:836-843.

5. Ruskin PE, Silver-Aylaian M, Kling MA, Reed SA, Bradham DD, Hebel JR, et al. (2004): Treatment outcomes in depression: comparison of remote treatment through telepsychiatry to in-person treatment. *Am J Psychiatry*. 161:1471-1476.

6. Elford R, White H, Bowering R, Ghandi A, Maddiggan B, St John K, et al. (2000): A randomized, controlled trial of child psychiatric assessments conducted using videoconferencing. *J Telemed Telecare*. 6:73-82.

7. Fortney JC, Pyne JM, Edlund MJ, Williams DK, Robinson DE, Mittal D, et al. (2007): A randomized trial of telemedicine-based collaborative care for depression. *J Gen Intern Med*. 22:1086-1093.

8. May C, Gask L, Atkinson T, Ellis N, Mair F, Esmail A (2001): Resisting and promoting new technologies in clinical practice: the case of telepsychiatry. *Soc Sci Med*. 52:1889-1901.

9. Frueh BC, Monnier J, Yim E, Grubaugh AL, Hamner MB, Knapp RG (2007): A randomized trial of telepsychiatry for post-traumatic stress disorder. *J Telemed Telecare*. 13:142-147.

10. Raue PJ, Schulberg HC, Heo M, Klimstra S, Bruce ML (2009): Patients' depression treatment preferences and initiation, adherence, and outcome: a randomized primary care study. *Psychiatr Serv*. 60:337-343.

11. Titov N, Dear BF, Johnston L, Lorian C, Zou J, Wootton B, et al. (2013): Improving adherence and clinical outcomes in self-guided internet treatment for anxiety and depression: randomised controlled trial. *PLoS One*. 8:e62873.

12. Dongier M, Tempier R, Lalinec-Michaud M, Meunier D (1986): Telepsychiatry: psychiatric consultation through two-way television. A controlled study. *Can J Psychiatry*. 31:32-34.

13. Burton C, Szentagotai Tatar A, McKinstry B, Matheson C, Matu S, Moldovan R, et al. (2016): Pilot randomised controlled trial of Help4Mood, an embodied virtual agent-based system to support treatment of depression. *J Telemed Telecare*. 22:348-355.

14. Cheng KM, Siu BW, Au Yeung CC, Chiang TP, So MH, Yeung MC (2018): Telepsychiatry for stable Chinese psychiatric out-patients in custody in Hong Kong: a case-control pilot study. *Hong Kong Med J*. 24:378-383.

15. Comer JS, Furr JM, Miguel EM, Cooper-Vince CE, Carpenter AL, Elkins RM, et al. (2017): Remotely delivering real-time parent training to the home: An initial randomized trial of Internet-delivered parent-child interaction therapy (I-PCIT). *J Consult Clin Psychol*. 85:909-917.

16. Crowe T, Jani S, Jani S, Jani N, Jani R (2016): A pilot program in rural telepsychiatry for deaf and hard of hearing populations. *Heliyon*. 2:e00077.

17. Iiboshi K, Yoshida K, Yamaoka Y, Eguchi Y, Sato D, Kishimoto M, et al. (2020): A Validation Study of the Remotely Administered Montreal Cognitive Assessment Tool in the Elderly Japanese Population. *Telemed J E Health*. 26:920-928.

18. Jones MD, Etherage JR, Harmon SC, Okiishi JC (2012): Acceptability and cost-effectiveness of military telehealth mental health screening. *Psychol Serv*. 9:132-143.

19. Khasanshina EV, Wolfe WL, Emerson EN, Stachura ME (2008): Counseling center-based tele-mental health for students at a rural university. *Telemed J E Health*. 14:35-41.

20. Modai I, Jabarin M, Kurs R, Barak P, Hanan I, Kitain L (2006): Cost effectiveness, safety, and satisfaction with video telepsychiatry versus face-to-face care in ambulatory settings. *Telemed J E Health*. 12:515-520.

21. Nelson EL, Barnard M, Cain S (2003): Treating childhood depression over videoconferencing. *Telemed J E Health*. 9:49-55.

22. Rohland BM (2001): Telepsychiatry in the heartland: if we build it, will they come? *Community Ment Health J*. 37:449-459.

23. Urness D, Wass M, Gordon A, Tian E, Bulger T (2006): Client acceptability and quality of life--telepsychiatry compared to in-person consultation. *J Telemed Telecare*. 12:251-254.

24. Ziemba SJ, Bradley NS, Landry LA, Roth CH, Porter LS, Cuyler RN (2014): Posttraumatic stress disorder treatment for Operation Enduring Freedom/Operation Iraqi Freedom combat veterans through a civilian community-based telemedicine network. *Telemed J E Health*. 20:446-450.
